# Loss of function of the nuclear envelope protein LEMD2 causes DNA damage–dependent cardiomyopathy

**DOI:** 10.1172/JCI158897

**Published:** 2022-11-15

**Authors:** Xurde M. Caravia, Andres Ramirez-Martinez, Peiheng Gan, Feng Wang, John R. McAnally, Lin Xu, Rhonda Bassel-Duby, Ning Liu, Eric N. Olson

**Affiliations:** 1 Department of Molecular Biology, Hamon Center for Regenerative Science and Medicine,; 2 Senator Paul D. Wellstone Muscular Dystrophy Specialized Research Center, and; 3 Quantitative Biomedical Research Center, Department of Population and Data Sciences and Department of Pediatrics, University of Texas Southwestern Medical Center, Dallas, Texas, USA.

**Keywords:** Cardiology, Cardiovascular disease, Molecular biology, Mouse models

## Abstract

Mutations in nuclear envelope proteins (NEPs) cause devastating genetic diseases, known as envelopathies, that primarily affect the heart and skeletal muscle. A mutation in the NEP LEM domain–containing protein 2 (LEMD2) causes severe cardiomyopathy in humans. However, the roles of LEMD2 in the heart and the pathological mechanisms responsible for its association with cardiac disease are unknown. We generated knockin (KI) mice carrying the human c.T38>G *Lemd2* mutation, which causes a missense amino acid exchange (p.L13>R) in the LEM domain of the protein. These mice represent a preclinical model that phenocopies the human disease, as they developed severe dilated cardiomyopathy and cardiac fibrosis leading to premature death. At the cellular level, KI/KI cardiomyocytes exhibited disorganization of the transcriptionally silent heterochromatin associated with the nuclear envelope. Moreover, mice with cardiac-specific deletion of *Lemd2* also died shortly after birth due to heart abnormalities. Cardiomyocytes lacking *Lemd2* displayed nuclear envelope deformations and extensive DNA damage and apoptosis linked to p53 activation. Importantly, cardiomyocyte-specific *Lemd2* gene therapy via adeno-associated virus rescued cardiac function in KI/KI mice. Together, our results reveal the essentiality of LEMD2 for genome stability and cardiac function and unveil its mechanistic association with human disease.

## Graphical abstract

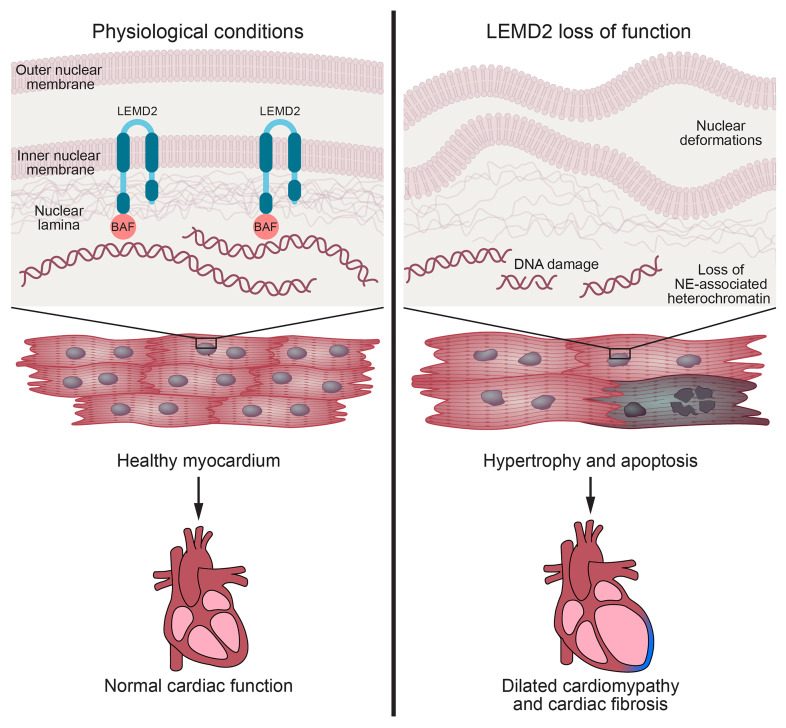

## Introduction

The nuclear envelope (NE) constitutes the boundary between the nucleus and the cytoplasm in eukaryotic cells. The inner (INM) and outer (ONM) nuclear membranes contain nuclear envelope proteins (NEPs) connected to the underlying nuclear lamina, a protein meshwork composed of lamin filaments that provide physical support for the entire structure (1). Collectively, NEPs execute a wide variety of essential cellular functions, such as mechanotransduction and chromatin organization (2). At least 80 NEPs have been identified in rodent liver (3), and several hundred are present in muscle cells (4, 5). The plethora of NEPs in muscle reflects their functional relevance in this tissue. To date, hundreds of mutations in lamins and NEPs have been shown to cause human pathological syndromes (6). Paradoxically, although lamins and several NEPs are ubiquitously expressed, their genetic mutations cause cardiac- or skeletal muscle–specific phenotypes in most cases (7). For example, mutations in the gene encoding the ubiquitously expressed NEP emerin cause a severe disease called Emery-Dreifuss muscular dystrophy (EDMD), which is characterized by skeletal muscle wasting and cardiac pathology (8–10). Various hypotheses have been proposed to explain the etiology of these pathologies collectively known as envelopathies (11). The mechanical stress hypothesis proposes that mutations in NEPs decrease the rigidity of the NE, affecting mechanotransduction and sensitizing cells to mechanical stress. This may explain why cardiac and skeletal muscles are particularly sensitive to alterations in NEPs. On the other hand, the gene expression hypothesis suggests that NEP mutations induce alterations in important signaling pathways and chromatin organization, which lead to aberrant gene expression patterns, causing pathological phenotypes. The lack of a complete understanding of the molecular basis of NE myopathies poses challenges for development of mutation-specific therapies for affected patients.

The lamina-associated polypeptide-emerin-MAN1 (LEM) family constitutes a group of proteins located in the INM that share the evolutionarily conserved LEM structural domain (12). LEM domain–containing protein 2 (LEMD2), which is expressed ubiquitously, is characterized by the presence of the LEM domain and 2 transmembrane domains. A series of in vitro studies revealed its ability to associate with DNA-binding proteins such as lamins and barrier-to-autointegration factor (BAF), which implicates LEMD2 as a mediator of the interaction between chromatin and the NE (13–15). Recent reports highlighted the requirement for LEMD2 in NE reformation after cell division as well as nuclear integrity and chromatin stabilization (16, 17). Notably, the first study in rodents regarding *Lemd2* showed that mice lacking this gene die during embryogenesis due to abnormal heart development (18). In humans, *LEMD2* mutations have also been linked to cardiac disease. Specifically, a *LEMD2* c.T38>G homozygous missense mutation is associated with arrhythmic cardiomyopathy, cataracts, and sudden death (19, 20). This nucleotide transversion (T38>G) results in the exchange of a leucine for an arginine in the LEM domain, which is essential for LEMD2 function (16). Together, these findings highlight the importance of LEMD2 for cardiac function even though its specific roles in the heart remain unknown.

Here, we generated a “humanized” *Lemd2* knockin (KI) mouse line carrying the same c.T38>G mutation found in patients and show that these animals develop systolic dysfunction and dilated cardiomyopathy (DCM) and die prematurely. We also show that cardiomyocytes (CMs) isolated from homozygous KI animals are hypertrophic and display a reduction in transcriptionally repressed heterochromatin associated with the NE. Cardiac-specific *Lemd2-*KO (cKO) mice also die shortly after birth due to cardiac abnormalities. Transcriptomic analysis in these 2 mouse models revealed strong activation of the p53 pathway. The aberrant activation of p53, a master regulator of genome integrity, is caused by extensive DNA damage triggered by LEMD2 loss of function, resulting in chronic activation of the DNA damage response (DDR) and apoptosis in *Lemd2* mutant mice. Immunostaining of isolated CMs lacking LEMD2 revealed nuclear deformations and abnormal mechanotransduction. Importantly, therapeutic delivery of the WT *Lemd2* specifically to CMs with adeno-associated virus (AAV) improved cardiac function of the KI/KI mice. Our findings highlight the important role of LEMD2 in cardiac homeostasis and provide mechanistic insights into the basis of LEMD2-associated cardiomyopathy.

## Results

### Generation and analysis of Lemd2 c.T38>G mice.

**Figure 1:**
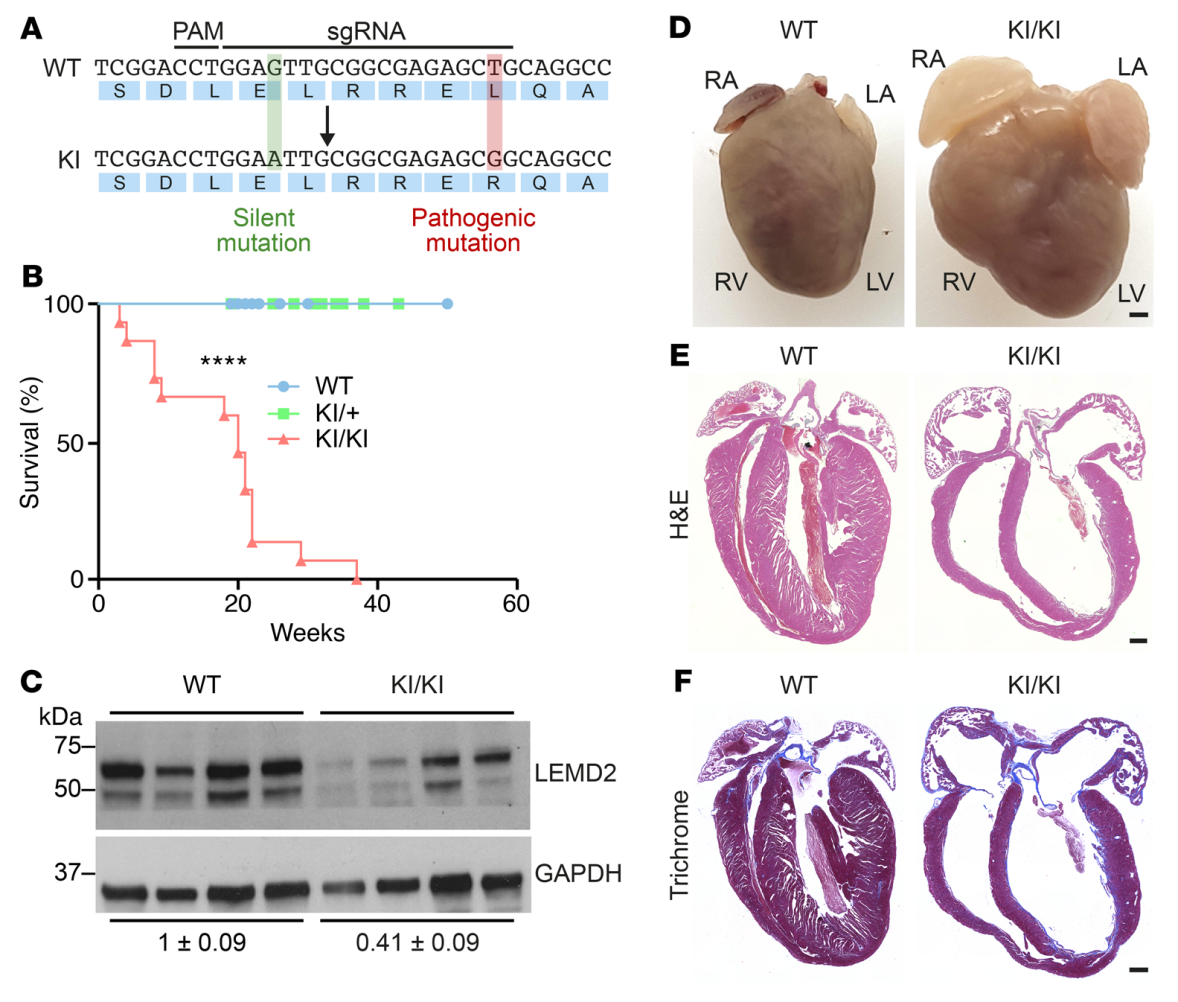
Cardiac abnormalities in KI/KI mice. (**A**) WT and *Lemd2* c.T38>G (KI) alleles showing the sgRNA and the protospacer adjacent motif (PAM) sequences as well as the introduced pathogenic (red) and silent (green) mutations. (**B**) Survival curve of WT (*n* = 17), KI/+ (*n* = 22), and KI/KI (*n* = 15) mice. ****P < 0.0001 for WT versus KI/KI comparison, log-rank (Mantel-Cox) test. (**C**) Western blot showing the levels of 2 cardiac LEMD2 protein isoforms in 2-month-old WT (*n* = 4) and KI/KI (*n* = 4) mice. Bottom: average and SEM of the relative LEMD2/GAPDH densitometry ratio in WT and KI/KI mice. (**D**) Macroscopic images of hearts from 3-month-old WT and KI/KI mice. Scale bar: 0.5 cm. RA, right atrium; LA, left atrium; RV, right ventricle. (**E**) H&E staining of hearts of 3-month-old WT and KI/KI mice. Scale bar: 500 μm. (**F**) Masson’s trichrome staining of 4-chamber view hearts from 3-month-old WT and KI/KI mice. Scale bar: 500 μm.

*Lemd2* mRNA is ubiquitously expressed across adult mouse tissues, with relatively higher expression in heart, skeletal muscle, liver, and kidney compared with lung and white adipose tissue (Supplemental Figure 1A; supplemental material available online with this article; https://doi.org/10.1172/JCI158897DS1). To investigate the molecular mechanisms underlying cardiomyopathy caused by the *LEMD2* c.T38>G mutation in humans (20), we generated mice carrying the same mutation, using CRISPR-Cas9 technology (Supplemental Figure 1B). Using a single-stranded oligonucleotide (ssODN) template, we changed nucleotide 38 within codon 13 of the coding region from thymine (T) to guanine (G) (c.T38>G), yielding a leucine-to-arginine substitution (Figure 1A). The mutation was confirmed by Sanger sequencing (Supplemental Figure 1C).

Homozygous *Lemd2* c.T38>G knockin (KI) mice, hereafter referred to as KI/KI, were born at expected Mendelian ratios from heterozygous crosses (Supplemental Figure 1D). However, while WT and heterozygous (KI/+) mice had normal longevity, we found that KI/KI mice died prematurely with a median life span of 20 weeks (Figure 1B). Similarly, patients carrying the same mutation suffer from sudden death at relatively young ages (between 30 and 50 years) (20). To determine whether the c.T38>G mutation affects *Lemd2* expression, we examined *Lemd2* expression at both RNA and protein levels in hearts of KI/KI mice. Quantitative reverse-transcription PCR (RT-qPCR) showed that *Lemd2* mRNA was modestly but significantly reduced in KI/KI mice compared with WT littermates (Supplemental Figure 1E). At the protein level, LEMD2 has 2 isoforms, expected to be at 57 and 30.7 KDa, respectively. Western blot analysis of hearts of WT animals revealed that both isoforms displayed a greater size, probably because of posttranslational modifications. Importantly, both isoforms were reduced (Figure 1C). To determine LEMD2 cellular localization, we overexpressed both WT and mutant forms of human LEMD2 using retroviral vectors in C2C12 myoblasts and differentiated them into myotubes. Confocal microscopy showed that both WT and mutant proteins closely associated with the nuclear periphery in myotubes (Supplemental Figure 1F). These results showed that LEMD2 protein with the c.T38>G (p.L13>R) mutation localizes similarly to WT LEMD2 protein. Interestingly, this mutant LEMD2 protein also showed normal localization in patient cardiac samples, although protein levels were not altered compared with those of controls (20).

Hearts from 3-month-old KI/KI mice revealed severe dilation of the atrial and ventricular chambers (Figure 1D). However, heart weight normalized to tibia length was similar in WT and KI/KI mice (Supplemental Figure 1G). H&E analysis revealed severe DCM in the KI/KI mice, characterized by cardiac chamber dilation and reduced ventricular wall thickness (Figure 1E). These pathological features indicate that the *Lemd2* c.T38>G mutation triggers severe cardiomyopathy in mice. Masson’s trichrome staining also showed cardiac fibrosis in the KI/KI mice (Figure 1F and Supplemental Figure 1H). A pattern of cardiac fibrosis was also detected in human patients carrying the same *LEMD2* mutation (20). We also performed histological examination of different skeletal muscle groups and observed no abnormalities in KI/KI mice (Supplemental Figure 1I). Skeletal muscle weight normalized to tibia length was similar in mice of both genotypes (Supplemental Figure 1J).

### KI/KI mice develop systolic dysfunction, DCM, and conduction system abnormalities.

**Figure 2:**
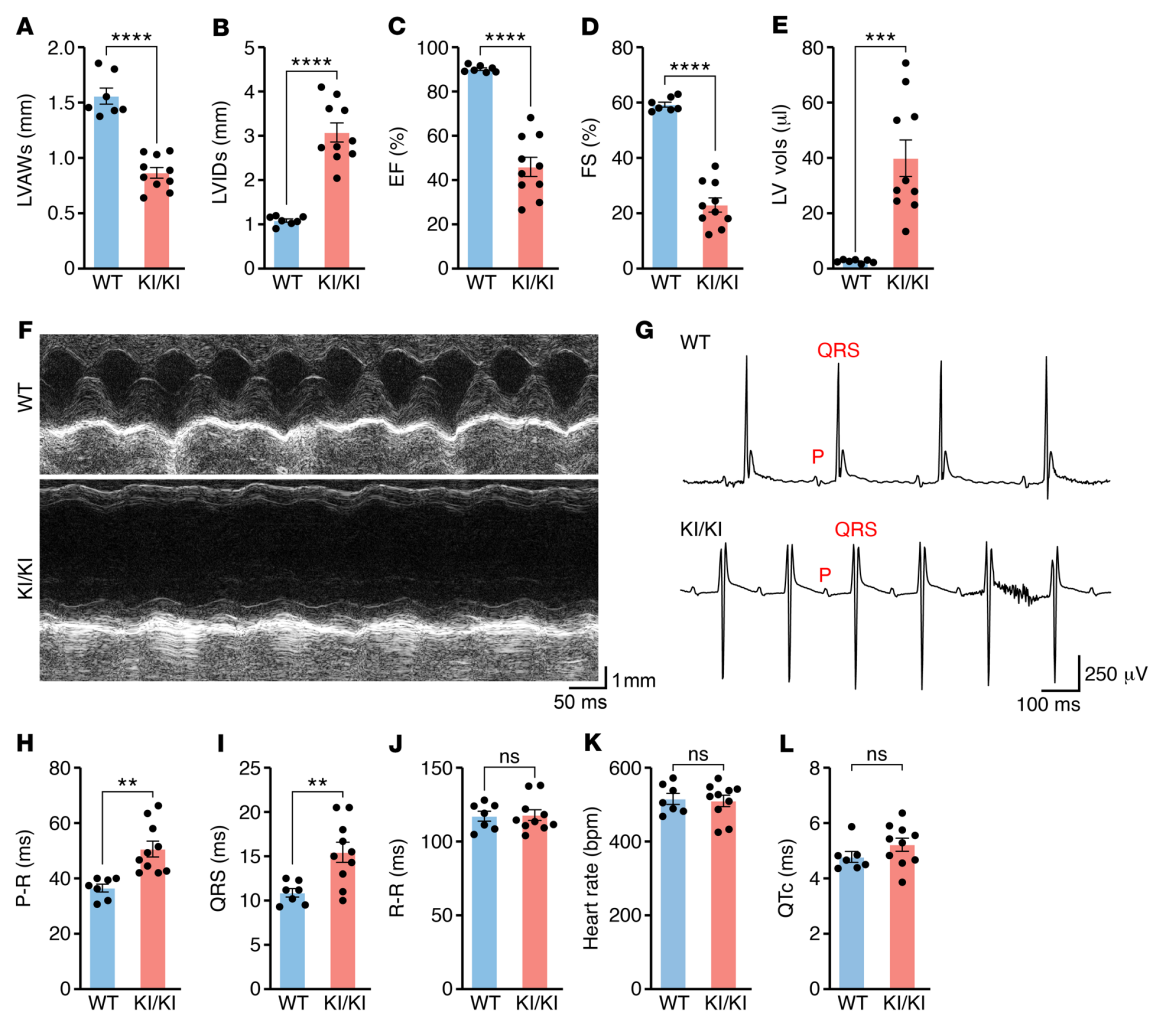
KI/KI mice develop systolic dysfunction and electrical abnormalities. Echocardiographic analysis of structural and functional parameters in systolic hearts from 2-month-old WT (*n* = 7) and KI/KI (*n* = 10) mice. (**A**) Systolic LVAW thickness, (**B**) systolic LVID, (**C**) EF, (**D**) FS, and (**E**) LV volume. ****P < 0.0001; ***P < 0.001, 2-tailed, unpaired *t* test. (**F**) Transthoracic M-mode echocardiographic tracings of 2-month-old WT and KI/KI mice. (**G**–**L**) EGC analysis of 2-month-old WT (*n* = 7) and KI/KI mice (*n* = 10). (**G**) Representative ECG of WT and KI/KI mice, (**H**) duration of P-R interval, (**I**) duration of QRS complex, (**J**) duration of R-R interval, (**K**) heart rate, and (**L**) duration of QTc interval. P-R, **P < 0.01; QRS, **P < 0.01; R-R, heart rate and QTc, *P* > 0.05; 2-tailed, unpaired *t* test.

The KI/KI mouse model recapitulates many important pathological phenotypes found in patients. Echocardiography also revealed that the KI/KI hearts showed a significant decrease in systolic left ventricular anterior wall (LVAW) thickness (Figure 2A) and a 3-fold increase in systolic left ventricular internal diameter (LVID) (Figure 2B). The ejection fraction (EF) of KI/KI mice was half that of WT mice (Figure 2C), and fractional shortening (FS) was also dramatically reduced (Figure 2D). Remarkably, the systolic left ventricular (LV) volume of KI/KI mice was on average 40 times greater than that of WT animals (Figure 2E), presumably as a result of impaired contractility (Figure 2F). Importantly, KI/KI mice developed these cardiac phenotypes early in life, since the main structural and functional cardiac parameters were significantly altered by 3 weeks after birth (Supplemental Figure 2, A–D). Conversely, the KI/+ mice were overtly normal and displayed completely preserved cardiac function (Supplemental Figure 3, A–G). These results indicate that the *Lemd2* c.T38>G mutation triggers pathological consequences specifically in the heart only when homozygous.

ECG revealed significant cardiac electrical alterations in KI/KI mice, characterized by an increased P-R interval, a hallmark of type I atrioventricular (AV) block (Figure 2, G and H). These results suggest a delay in the conduction of the electrical signal from the atria to the ventricles through the AV node. The duration of the QRS complex, which corresponds to ventricle depolarization, was also augmented in KI/KI animals (Figure 2I). Although type I AV block is not a life-threatening condition, we found that these abnormalities worsened to a type II AV block in a subset of KI/KI mice, in which P waves were not followed by a QRS complex, (Supplemental Figure 4A). We did not detect alterations in cardiac rhythm or in the corrected QT (QTc) duration, an indicator of cardiac arrhythmias, in KI/KI mice under basal conditions (Figure 2, J–L). To further determine the cause of premature death, we subjected KI/KI mice to acute β-adrenergic stimulation using isoproterenol (ISO) and recorded the ECG for 35 minutes (Supplemental Figure 4B). KI/KI mice died before the end of the experiment after showing severe AV blocks, suggesting that cardiac electrical alterations play an important role in the reduced longevity of the KI/KI mice (Supplemental Figure 4, C and D). Finally, to rule out any intrinsic alterations in the AV node, we performed immunostaining for the potassium channel HCN4, a specific marker of the cardiac conduction system (CCS), and corroborated that the morphology and size of the AV node were similar in WT and KI/KI mice, suggesting that the electrical alterations were not primarily caused by defects in the CCS (Supplemental Figure 4E). We speculate that fibrosis accumulation in the heart could account for these alterations in cardiac conduction. Overall, these observations suggest that KI/KI mice die prematurely due to cardiac abnormalities. Moreover, these findings confirmed the histological analysis and demonstrated severe DCM and systolic dysfunction in KI/KI mice, resembling the pathological features of patients carrying the same mutation, who also display a reduction in EF and electrical abnormalities (20).

### Chromatin abnormalities and transcriptomic alterations in KI/KI mice.

**Figure 3:**
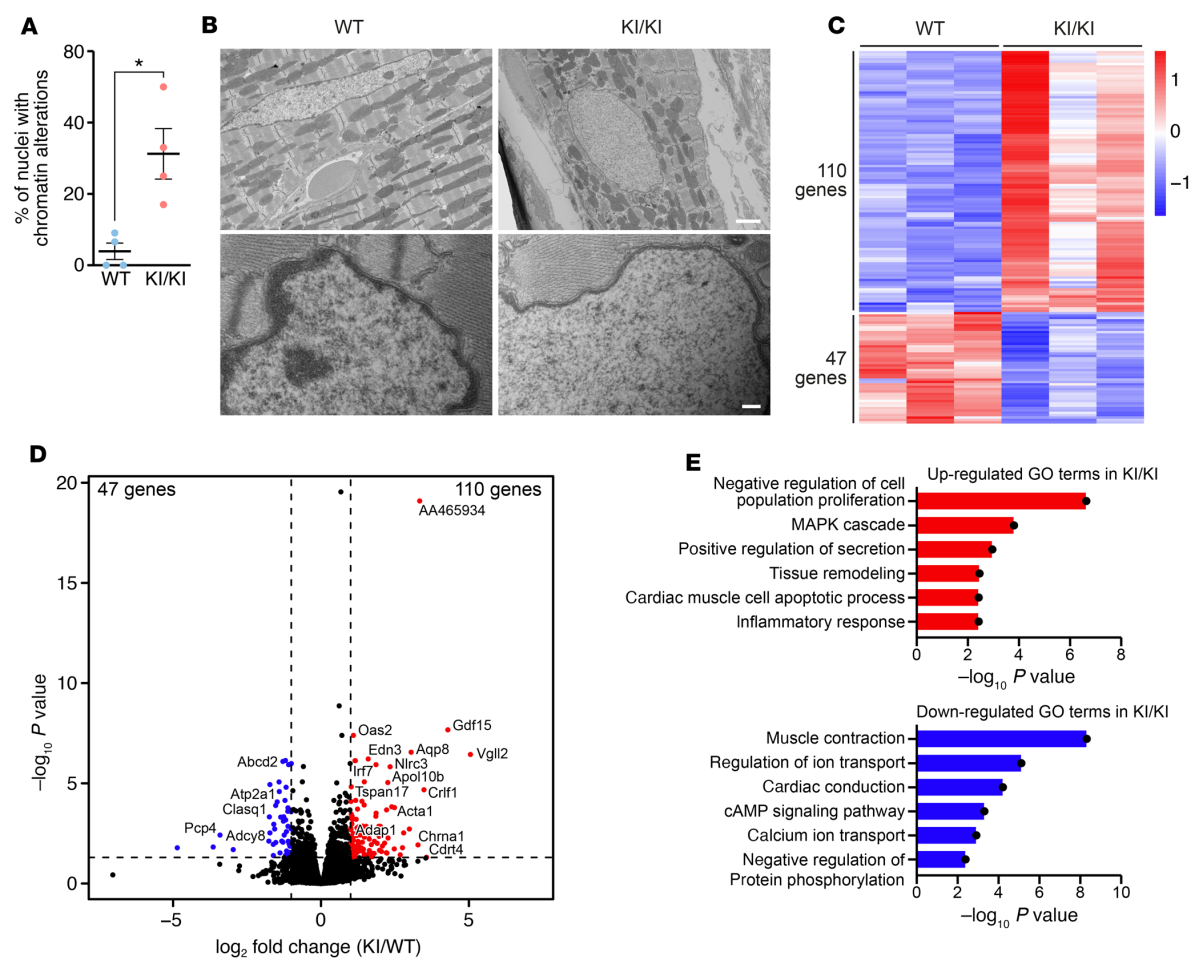
Chromatin and transcriptomic alterations in KI/KI mice. (**A**) Percentage of nuclei with reduction in NE-associated heterochromatin. *n* = 4 mice per genotype. *P < 0.05, 2-tailed, unpaired *t* test. (**B**) Representative electron microscopy photographs of 3-month-old WT and KI/KI nuclei. Scale bars: 2 μm (top); 200 nm (bottom). (**C**) Heatmap showing the DE genes in WT and KI/KI mice. *n* = 3 mice per genotype. Expression level in *Z*-score, FDR-adjusted *P* of 0.05 was used as cutoff, Benjamini–Hochberg method. (**D**) Volcano plot showing fold-change and *P* values of genes upregulated (red) and downregulated (blue) in KI/KI compared with WT mice. *n* = 3 mice per genotype. FDR-adjusted *P* < 0.05 and fold-change >1.5, Benjamini–Hochberg method. (**E**) GO terms up- and downregulated in KI/KI mice compared with WT animals. *n* = 3 mice per genotype. *P* < 0.01.

To gain mechanistic insights into LEMD2-associated cardiomyopathy, we analyzed the global chromatin organization of the heart by electron microscopy. The repositioning of chromatin close to the NE is an important mechanism for controlling gene expression, and this process is largely controlled by NEPs, including LEMD2 (21). The electron-dense NE-associated heterochromatin in WT hearts was readily apparent. However, this heterochromatin was almost absent in more than 30% of the KI/KI nuclei (Figure 3, A and B). Thus, these findings indicate that LEMD2 plays a role in chromatin organization in the heart that is impaired by the c.T38>G mutation in the LEM domain.

The loss of NE-associated heterochromatin is expected to cause derepression of silenced genes in mutant mice. To explore this possibility and to further understand the molecular basis of cardiac dysfunction in mutant hearts, we performed RNA-Seq of hearts from 2-month-old WT and KI/KI mice. This analysis revealed 110 upregulated and 47 downregulated genes in KI/KI mice compared with their WT littermates (Figure 3, C and D). Gene Ontology (GO) analysis showed activation of pathways that impair cellular proliferation and promote hypertrophy (Figure 3E). Among these, the MAPK pathway, which has been associated with CM hypertrophy and extracellular matrix remodeling in the heart (22), was enriched in KI/KI hearts. Accordingly, genes related to tissue remodeling also showed enriched expression in KI/KI hearts. In this regard, it has been reported that *Lemd2* global KO mice also show strong activation of the MAPK pathway, including an increase in ERK1/2, JNK, and p38α phosphorylation measured in protein extracts from E10.5 embryos (18). On the other hand, we found repression of pathways related to calcium signaling and muscle function, including muscle contraction, as well as repression of genes associated with cardiac conduction, consistent with the alteration of cardiac conduction in KI/KI mice (Figure 3E). Among the most upregulated genes was *Gdf15*, a member of the TGF-β family that is not expressed in the healthy heart, but is induced by p53 signaling as a stress response after hypertrophy or DCM (23, 24). Another gene, Adap1, which encodes the GTPase-activating protein ArfGAP with dual PH domain 1 and is linked to cardiac hypertrophy, was also upregulated (25). Conversely, the calmodulin-signaling pathway regulator *Pcp4a* and the adenylyl cyclase *Adcy8*, which regulate cardiac rhythmicity, were downregulated in the KI/KI animals (Figure 3D) (26, 27). Taken together, these results highlight the activation of prohypertrophic and antiproliferative pathways in the KI/KI mice that could explain the cardiac abnormalities in the mutant animals.

### Hypertrophy and DNA damage in KI/KI hearts.

**Figure 4:**
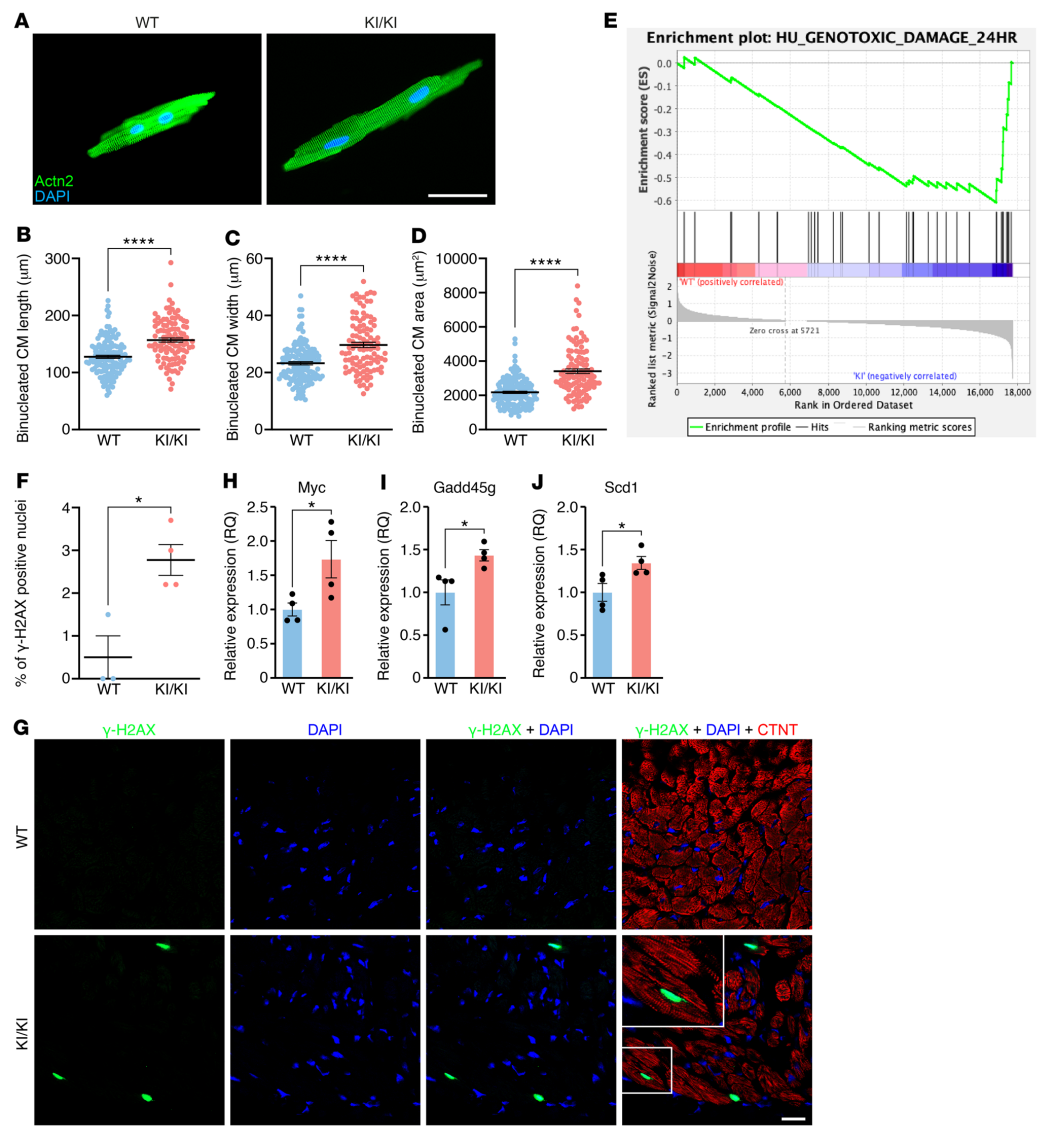
CM hypertrophy and DNA damage in KI/KI mice. (**A**) Representative images of isolated CMs from 3-month-old WT and KI/KI mice. Scale bar: 50 μm. (**B**) Length of binucleated CMs isolated from 3-month-old WT and KI/KI mice. *n* = 3–4 mice per genotype, 100–150 total cells per genotype. ****P < 0.0001, 2-tailed, unpaired *t* test. (**C**) Width of binucleated CMs isolated from 3-month-old WT and KI/KI mice. *n* = 3–4 mice per genotype, 100–150 total cells per genotype. ****P < 0.0001, 2-tailed, unpaired *t* test. (**D**) Area of binucleated CMs isolated from 3-month-old WT and KI/KI mice. *n* = 3–4 mice per genotype, 100–150 total cells per genotype. ****P < 0.0001, 2-tailed, unpaired *t* test. (**E**) GSEA plot showing enrichment of genes related to genotoxic damage in KI/KI mice. Note that the enrichment score (green line) deviates from 0 in the right part of the plot, indicating that those genes are enriched in the KI/KI mice. *n* = 3 mice per genotype. (**F**) Percentage of nuclei positive for γ-H2AX staining in WT and KI/KI mice. *n* = 3–4 mice per genotype, more than 100 nuclei per mouse. *P < 0.05, 2-tailed, unpaired *t* test. (**G**) Representative photographs of γ-H2AX and cTNT staining in cardiac sections from WT and KI/KI mice. Scale bar: 20 μm. Note that the white square part of the bottom left panel has been zoomed in. Original magnification, ×160. (**H**–**J**) Cardiac mRNA expression of genes related to p53 signaling and DDR in WT and KI/KI. *n* = 4 mice per genotype. Myc, *P < 0.05; Gadd45g, *P < 0.05; Scd1, *P < 0.05, 2-tailed, unpaired *t* test.

To understand the cellular consequences of the transcriptional alterations in KI/KI hearts, we isolated CMs from 2-month-old WT and KI/KI hearts and performed immunostaining for the sarcomere protein α-actinin (ACTN2) to measure CM size and morphology (Figure 4A). CMs isolated from KI/KI hearts showed significant increases in length, width, and area compared with WT CMs (Figure 4, B–D). We also subjected isolated CMs to an electrical stimulator (pacing) to study their contractility and calcium handling (Supplemental Figure 5A). This assay revealed that the length of sarcomeres as well as their FS upon electrical stimulation were normal (Supplemental Figure 5, B and C). Additionally, using the fluorescent dye Fura-2, we observed that the diastolic calcium levels, the transient amplitude, and the time to calcium peak were also preserved in KI/KI CMs (Supplemental Figure 5, D–F). Furthermore, transmission electron microscopy (TEM) on cardiac sections revealed normal sarcomere ultrastructure in the KI/KI mice (Supplemental Figure 5G).

To further understand the pathogenic mechanisms of LEMD2-associated cardiomyopathy, we performed Gene Set Enrichment Analysis (GSEA) on cardiac genes differentially expressed (DE) between WT and KI/KI mice. Among the pathways significantly enriched in the KI/KI mice, we found a set of genes related to genotoxic stress, suggesting that DNA damage might play a role in the observed phenotype of the KI/KI mice (Figure 4E). To confirm this observation, we performed immunofluorescence analysis of the γ-phosphorylation of Ser139 of histone H2AX, a well-known marker of DNA double-strand break (28). We found a greater than 3-fold increase in the number of γ-H2AX–positive nuclei in cardiac sections of KI/KI mice compared with WT littermates (Figure 4F). The number of double-strand breaks, evidenced by γ-H2AX staining, was readily apparent in KI/KI hearts (Figure 4G). Additionally, we performed RT-qPCR for genes related to DNA damage. We found that *Myc*, an activator of the DNA damage–dependent p53 pathway, and the *Gadd45g* and *Scd1* genes, involved in the DDR, were significantly upregulated in KI/KI hearts (Figure 4, H–J). These findings suggest the presence of genotoxic stress and DNA damage in KI/KI hearts.

### Lemd2 cardiac deficiency leads to cardiomyopathy and premature death in mice.

KI/KI mice carry a global hypomorphic mutation, preventing the analysis of cardiac-specific functions of LEMD2. To explore the functions of LEMD2 specifically in CMs, we generated a conditional allele of *Lemd2* by introducing 2 loxP sites flanking the first exon of the *Lemd2* gene using CRISPR-Cas9 gene editing, hereafter referred to as *Lemd2^fl/fl^* (Supplemental Figure 6A). We then bred this mouse line with Myh6-Cre transgenic mice expressing Cre recombinase under the control of the cardiac α-myosin heavy chain (α-MHC) promoter, to enable cardiac-specific deletion of *Lemd2* (cKO) (29). We confirmed the excision of the floxed alleles (Supplemental Figure 6B) and the reduction in both LEMD2 protein isoforms in hearts from cKO animals compared with those from *Lemd2^fl/fl^* mice (Supplemental Figure 6C). We attribute residual expression of LEMD2 in cardiac extracts to non-CMs, which make up approximately half of the cells in the heart (30).

**Figure 5:**
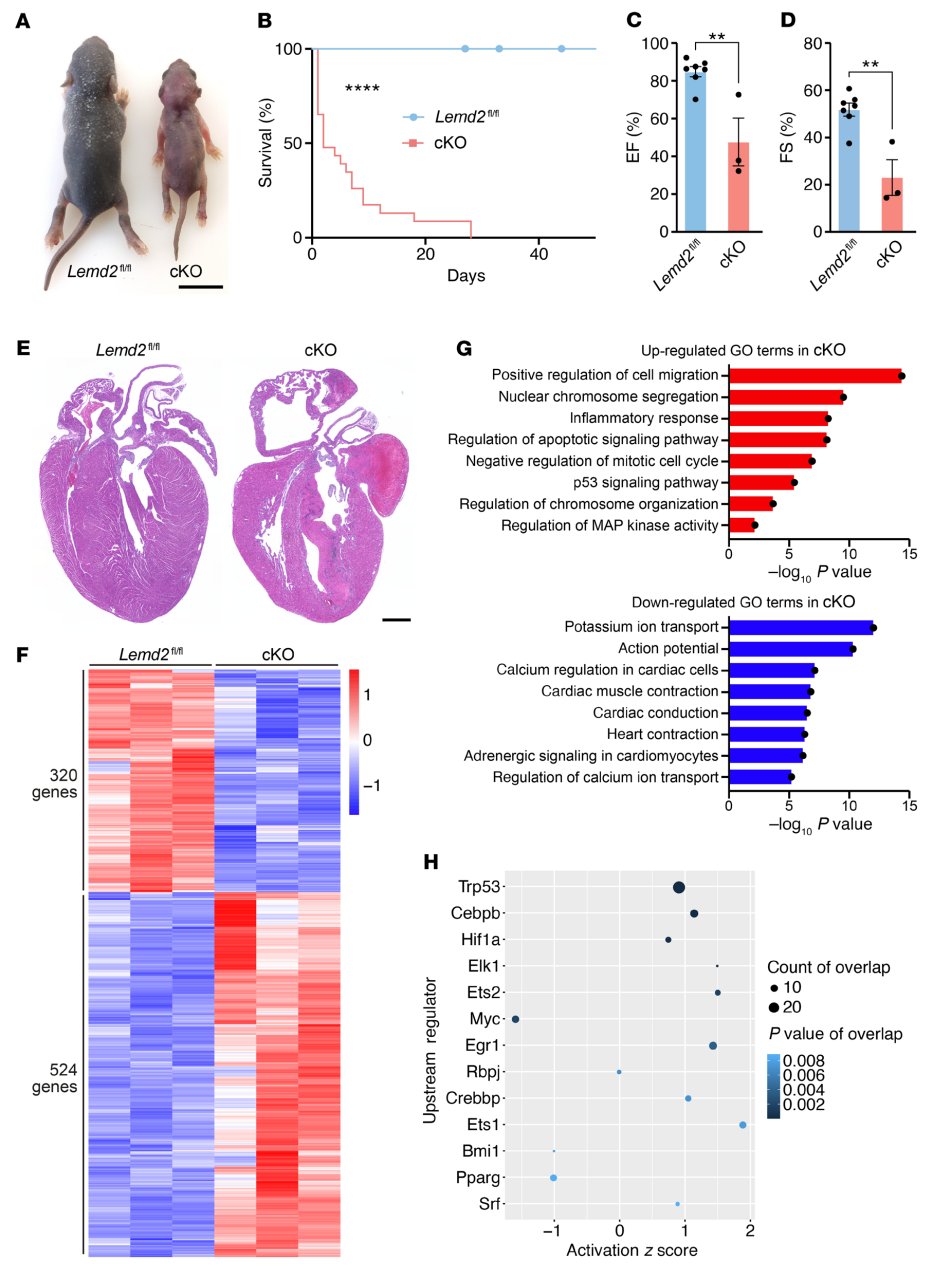
*Lemd2* deficiency in the heart leads to cardiomyopathy and premature death in mice. (**A**) Representative photograph of *Lemd2^fl/fl^* (left) and cKO (right) mice at P7. Scale bar: 1 cm. (**B**) Survival curve of *Lemd2^fl/fl^* (*n* = 19) and cKO (*n* = 23) mice. ****P < 0.0001, log-rank (Mantel-Cox) test. (**C**) Echocardiographic analysis of systolic EF of hearts from *Lemd2^fl/fl^* (*n* = 7) and cKO (*n* = 3) mice (from 2 to 10 postnatal days).**P < 0.01, 2-tailed, unpaired *t* test. (**D**) Echocardiographic analysis of systolic FS of hearts from *Lemd2^fl/fl^* (*n* = 7) and cKO (*n* = 3) mice (from 2 to 10 postnatal days). **P < 0.01, 2-tailed, unpaired *t* test. (**E**) H&E staining of 4-chamber view P7 hearts from *Lemd2^fl/fl^* and cKO mice. Scale bar: 500 μm. Note the atrial thrombi in cKO hearts. (**F**) Heatmap showing the DE genes in *Lemd2^fl/fl^* and cKO mice. *n* = 3 mice per genotype. Expression level in *Z*-score. FDR-adjusted *P* value of 0.05 was used as cutoff, Benjamini–Hochberg method. (**G**) Enriched GO terms up- and downregulated in cKO compared with *Lemd2^fl/fl^* mice. *n* = 3 mice per genotype. *P* < 0.01. (**H**) Upstream regulator analysis of DE genes in cKO mice compared with *Lemd2^fl/fl^* mice. *n* = 4 mice per genotype. Activation in *Z*-score.

cKO mice were born at Mendelian ratios (Supplemental Figure 6D), but developed a striking postnatal phenotype, characterized by a reduction in body size immediately after birth and neonatal lethality, with 50% mortality of cKO mice by 2 days of age (Figure 5, A and B). Echocardiographic analysis revealed that cKO mice showed a significant reduction in both EF and FS compared with *Lemd2^fl/fl^* littermates within the first 10 days of life (Figure 5, C and D). cKO mice displayed systolic dysfunction with severely impaired contraction of the LV (Supplemental Figure 6E). Histological analysis showed dilation of atrial and ventricular chambers in cKO mice (Figure 5E). The finding that *Lemd2* mice developed a stronger cardiac phenotype than KI/KI mice suggests that the c.T38>G mutation yields a hypomorphic protein with partially compromised function. We therefore used the cKO animals to study the molecular consequences of *Lemd2* loss of function in the heart.

We performed transcriptomic analysis by RNA-Seq on cardiac samples from P1 *Lemd2^fl/fl^* and cKO mice and identified 844 DE genes in cKO hearts (Figure 5F). GO analysis revealed that the most upregulated pathways were related to apoptosis and negative regulators of proliferation and cell cycle progression (Figure 5G). Conversely, pathways related to cardiac performance, including cardiac conduction, heart contraction, and calcium regulation, were downregulated in cKO hearts. Overall, the transcriptomic dysregulation of the cKO hearts strongly resembled the alterations found in the KI/KI hearts, suggesting that common molecular mechanisms could drive the development of cardiomyopathy in both mouse models.

Interestingly, we also found significant enrichment of pathways related to chromatin organization and activation of the p53-signaling pathway in hearts of cKO mice (Figure 5G). p53 Regulates the expression of many genes related to apoptosis, senescence, and the DDR (31, 32). Upstream regulator analysis of the DE genes identified transcription factors that drive the expression of genes altered in cKO mice; p53 was at the top of the list, controlling the expression of 29 genes, consistent with the activation of the p53-signaling pathway in cKO mice (Figure 5H). Overall, these results demonstrate that LEMD2 is an essential protein for cardiac homeostasis and function and that its loss of function leads to severe cardiomyopathy in mice with activation of the DNA-damage response and p53-dependent gene expression.

### NE deformations, DNA damage, and cellular apoptosis in cKO mice.

To explore the mechanistic underpinnings of the cardiac abnormalities in cKO mice, we performed GSEA and found that the p53 downstream pathway was highly enriched in cKO hearts (Supplemental Figure 7A). We also identified numerous genes belonging to the p53-signaling pathway and involved in the DDR as being dysregulated in cKO hearts, as measured by RT-qPCR (Supplemental Figure 7B). To validate these results, we performed immunostaining for γ-H2AX on cardiac sections from 5-day-old (P5) *Lemd2^fl/fl^* and cKO mice. Histochemical analysis revealed that, while *Lemd2^fl/fl^* nuclei showed minimal damage, nuclei from cKO hearts were severely affected, with 6% of the total nuclei positive for H2AX phosphorylation (Supplemental Figure 7, C and D). Interestingly, we also found a significant decrease in cellular proliferation, measured by the Ki67 marker (Supplemental Figure 7, E and F). Finally, we performed TUNEL staining to detect cellular apoptosis in cKO hearts. This analysis revealed an increase in apoptotic cells in cKO compared with *Lemd2^fl/fl^* hearts (Supplemental Figure 7, G and H). The reduction in proliferation and the increase in apoptosis could be, at least in part, a direct consequence of high DNA damage. Thus, the percentage of apoptotic nuclei was almost the same as the percentage of cells that showed DNA damage, suggesting that chronic DNA damage triggers cell death in cKO mice.

**Figure 6:**
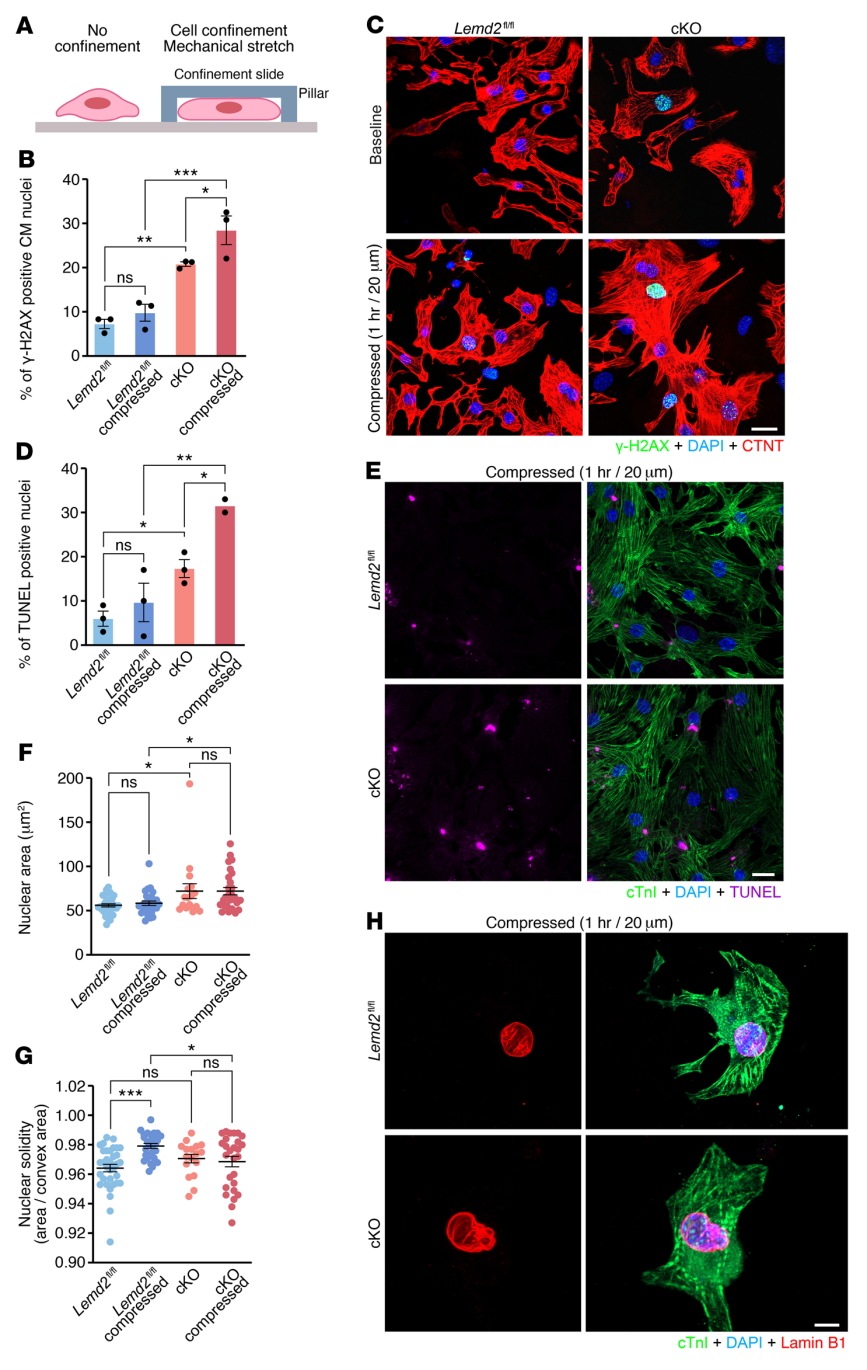
DNA damage and cellular apoptosis in cKO mice. (**A**) Schematic representation of the confiner device. (**B**) Percentage of nuclei positive for γ-H2AX in *Lemd2^fl/fl^* and cKO CMs isolated from P1 mice at baseline and after 20 μm compression for 1 hour. Three experimental replicates. *n* = 2–5 mice per genotype, more than 80 nuclei per replicate. ***P < 0.001; **P < 0.01; *P < 0.05, 1-way ANOVA and Holm-Šidák test for correction of multiple comparisons. (**C**) Representative images of γ-H2AX and cTNT staining in CMs isolated from P1 *Lemd2^fl/fl^* and cKO mice at baseline and after 20 μm compression for 1 hour. Scale bar: 20 μm. (**D**) Percentage of TUNEL-positive nuclei in *Lemd2^fl/fl^* and cKO CMs isolated from P1 mice at baseline and after 20 μm compression for 1 hour. 2–3 replicates. *n* = 2–5 mice per genotype, more than 80 nuclei per replicate. **P < 0.01; *P < 0.05, 1-way ANOVA and Holm-Šidák test for correction of multiple comparisons. (**E**) Representative images of TUNEL and cTnI staining in *Lemd2^fl/fl^* and cKO CMs isolated from P1 mice and compressed at 20 μm for 1 hour. Scale bar: 20 μm. (**F**) Nuclei area in CMs isolated from P1 *Lemd2^fl/fl^* and cKO mice at baseline and after 20 μm compression for 1 hour. *n* = 2 mice per genotype, 15–30 nuclei per genotype. *P < 0.05, 1-way ANOVA and Holm-Šidák test for correction of multiple comparisons. (**G**) Nuclear solidity (area/convex area) in CMs isolated from P1 *Lemd2^fl/fl^* and cKO mice at baseline and after 20 μm compression for 1 hour. *n* = 2 mice per genotype, 15–30 nuclei per genotype. ***P < 0.001; *P < 0.05, 1-way ANOVA and Holm-Šidák test for correction of multiple comparisons. (**H**) Representative images of lamin B1 and cTnI staining in *Lemd2^fl/fl^* and cKO CMs isolated from P1 mice and compressed at 20 μm for 1 hour. Scale bar: 5 μm.

To further validate the causal link between LEMD2 deficiency and DNA damage, we determined whether LEMD2 also participates in NE stability and mechanotransduction, an important cellular process that senses internal and external mechanical forces and allows cells to respond (33). We isolated CMs from hearts of *Lemd2^fl/fl^* and cKO mice at P1 and subjected them to mechanical stretching using a confiner device (Figure 6A) (34). Importantly, we validated our previous results, since under basal conditions, cKO CMs exhibited higher levels of DNA damage than those isolated from *Lemd2^fl/fl^* hearts (Figure 6, B and C). Subsequently, when we subjected these cells to 20 μm confinement for 1 hour, we observed that *Lemd2^fl/fl^* CMs showed DNA damage levels similar to those of uncompressed cells. Conversely, mechanical stress triggered exacerbated DNA damage in cKO CMs (Figure 6, B and C). Accordingly, TUNEL staining on the same cells revealed a very similar pattern, strongly suggesting that NE instability leads to cellular apoptosis in cKO hearts (Figure 6, D and E). These results indicate that, at least in cardiac cells, the LEMD2 protein participates in organizing and stabilizing chromatin under mechanical stress.

Finally, we investigated the occurrence of NE deformations as a potential pathogenic mechanism and source of DNA damage. We stained CMs from hearts of *Lemd2^fl/fl^* and cKO P1 mice for the NEP lamin B1 and subjected them to mechanical stress. We noticed that *Lemd2*-deficient nuclei were bigger than *Lemd2^fl/fl^* both at baseline and after compression, which suggests nuclear instability and alterations in chromatin organization (Figure 6F). To further quantify nuclear deformations, we performed morphometric analysis of the isolated nuclei by calculating their solidity, an indicator of nuclear blebbing. We observed no differences between *Lemd2^fl/fl^* and cKO nuclei under basal conditions. However, while control nuclei were able to adapt their morphology to compression by increasing their solidity, *Lemd2*-deficient CMs failed to adapt to the mechanical stress and showed blebs, suggesting that LEMD2 plays a role in adaptation to mechanical stress (Figure 6, G and H). Taken together, these findings show that *Lemd2* deficiency renders the NE more susceptible to deformations under mechanical stress, which in turns generates DNA damage and cellular apoptosis in cKO CMs.

### Lemd2 gene therapy improves cardiac function in KI/KI mice.

**Figure 7:**
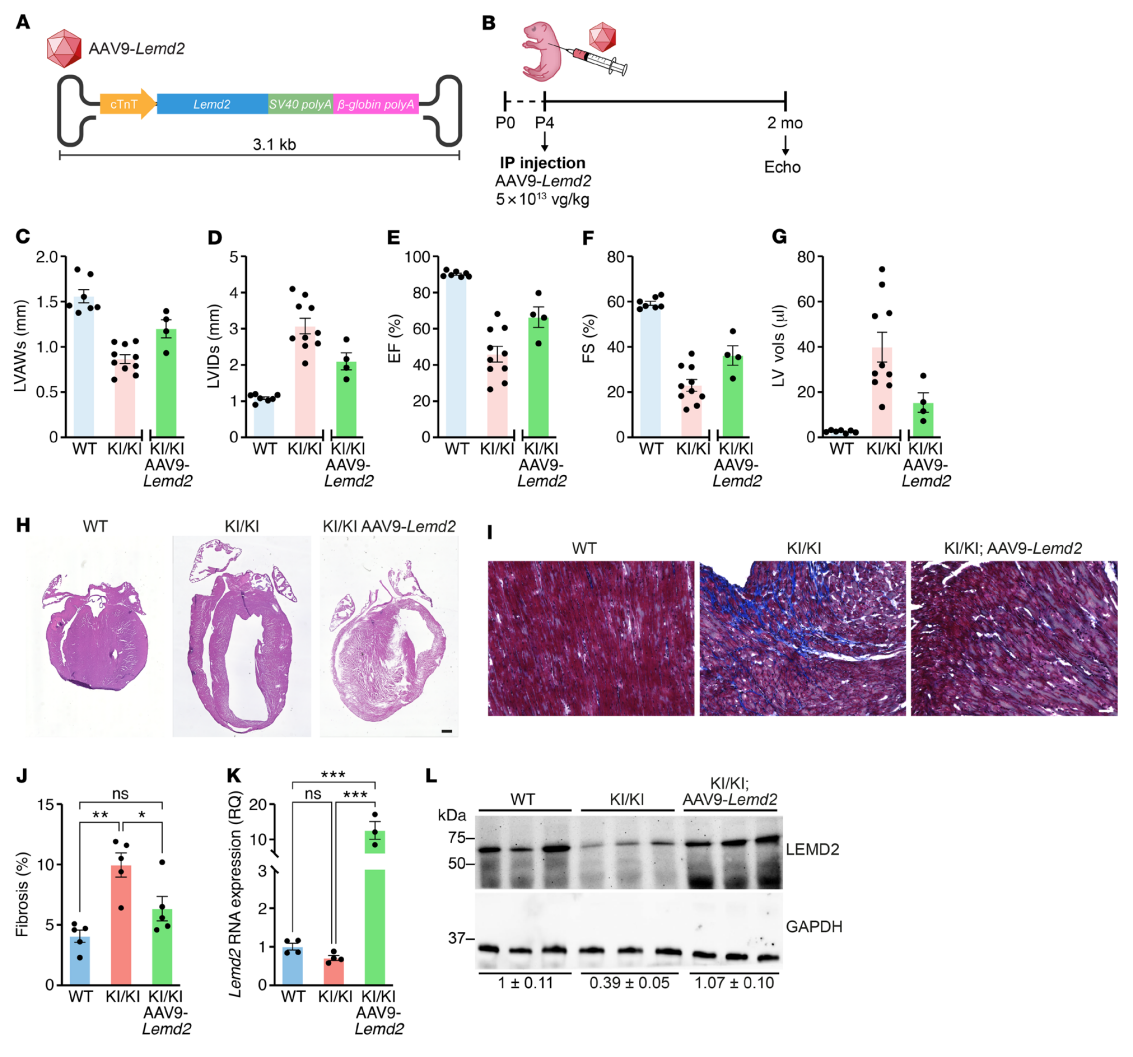
*Lemd2* gene therapy improves cardiac function in KI/KI mice. (**A**) Schematic of the AAV9-*Lemd2* system for in vivo delivery. (**B**) Overview of the in vivo injection strategy. (**C**–**G**) Echocardiographic analysis of structural and functional parameters in hearts from 2-month-old WT (*n* = 7), KI/KI (*n* = 10), and KI/KI AAV9-*Lemd2* mice (*n* = 4). The AAV9-*Lemd2* treatment experiment was unblinded for mouse genotypes, and data are compared with untreated WT and KI/KI groups shown in Figure 2, A–F, that were not assessed contemporaneously. (**C**) Systolic LVAW thickness, (**D**) systolic LVID, (**E**) EF, (**F**) FS, and (**G**) LV volume. (**H**) H&E staining of 4-chamber view of 3-month-old hearts from WT, KI/KI, and KI/KI AAV9-*Lemd2* mice. Scale bar: 500 μm. (**I**) Masson trichrome staining of 3-month-old hearts from WT, KI/KI, and KI/KI AAV9-*Lemd2* mice. Scale bar: 50 μm. (**J**) Quantification of the percentage of cardiac fibrosis in hearts from WT, KI/KI, and KI/KI AAV9-*Lemd2* mice. 4–5 cardiac sections per mouse. *n* = 1 mouse per genotype. **P < 0.01; *P < 0.05, 1-way ANOVA and Holm-Šidák test for correction of multiple comparisons. (**K**) *Lemd2* mRNA expression in hearts from 2-month-old WT (*n* = 4), KI/KI (*n* = 4), and KI/KI AAV9-*Lemd2* (*n* = 3) mice. ***P < 0.001, 1-way ANOVA and Holm-Šidák test for correction of multiple comparisons. (**L**) Western blot showing the levels of 2 cardiac LEMD2 protein isoforms in 2-month-old WT (*n* = 3), KI/KI (*n* = 3), and KI/KI AAV9-*Lemd2* (*n* = 3) mice. Bottom: average and SEM of the relative LEMD2/GAPDH densitometry ratio in WT, KI/KI, and KI/KI AAV9-*Lemd2* mice.

The severity of the LEMD2-associated cardiomyopathy in humans highlights the need for therapeutic approaches aimed at targeting the pathogenic cause of the disease. In this regard, since the c.T38>G mutation causes reduction in LEMD2 mutant protein levels, we hypothesized that an increase in the expression level of the WT full-length LEMD2 protein could provide therapeutic benefits. To test this hypothesis, we engineered AAV serotype 9 (AAV9), which displays cardiac tropism, to express the mouse *Lemd2* gene under the cardiac-specific promoter of the troponin T (*cTnT*) gene (Figure 7A). AAV9 was delivered i.p. to mice at P4 at a dose of 5 × 10^13^ vg/kg, and echocardiography was performed 2 months later (Figure 7B). Data were compared with the reference values shown in Figure 2, A–F. KI/KI mice treated with AAV9-*Lemd2* showed an increase in systolic LVAW thickness and a smaller LVID compared with KI/KI untreated mice (Figure 7, C and D). Moreover, we found an improvement in numerous cardiac functional parameters of the AAV9-treated KI/KI mice, including EF, FS, and LV volume, compared with that in untreated KI/KI animals (Figure 7 E–G). Histological analysis corroborated that the DCM phenotype and the fibrotic accumulation were substantially ameliorated (Figure 7, H–J). At the molecular level, *Lemd2* mRNA levels were upregulated more than 10-fold in the hearts of KI/KI mice after AAV treatment compared with that in WT animals (Figure 7K). However, protein levels after AAV9-*Lemd2* delivery were very similar to those of WT mice, indicating a rescue of the protein expression to physiological levels (Figure 7L).

## Discussion

This is the first study, to our knowledge, to explore the role of LEMD2, a ubiquitous NEP, in heart disease and cardiac development. Our findings reveal the essentiality of LEMD2 for cardiac homeostasis and proper heart function. Mice carrying the same *Lemd2* mutation found in humans (c.T38>G) recapitulate the main pathological features of patients with this mutation, including impaired heart function, cardiac fibrosis, and premature sudden death (20). In this regard, the KI/KI mice represent a valuable tool for studying LEMD2-associated cardiomyopathy and can be utilized to unravel the molecular mechanisms of this condition and to provide a preclinical model for testing potential therapies. Furthermore, to study LEMD2 function specifically in CMs, we generated the CM-specific cKO mouse model. These animals display a stronger cardiac phenotype than KI/KI mice, resulting in a median survival of 2 days. Our findings further highlight the importance of LEMD2 for normal cardiac function. Additionally, the perinatal lethality of cKO mice suggests that LEMD2 may also be necessary for successful completion of cardiac maturation. Importantly, embryos with a global deletion of *Lemd2* die at E11.5, presumably due to cardiac abnormalities (18). In summary, both KI/KI and cKO mice display a phenotype characterized by systolic dysfunction and DCM.

Our data strongly suggest that the *Lemd2* c.T38>G mutation is a recessive hypomorphic mutation that causes a reduction in gene expression or protein stability. Thus, the mutant LEMD2 protein is substantially reduced compared with that of WT animals, which triggers the pathological consequences. This conclusion is reinforced by the fact that the KI/+ heterozygous mice are normal. Moreover, this also explains why the cKO mice, in which LEMD2 is completely absent in CMs, develop more severe disease. We conclude that a threshold level of LEMD2 is required for normal cardiac function and that its loss leads to cardiomyopathy.

Alterations in other NEPs, such as emerin, another ubiquitously expressed and highly conserved LEM-containing protein, also produce strong phenotypes in both heart and skeletal muscle (8). Thus, recessive loss of emerin leads to X-linked EDMD, a severe envelopathy characterized by progressive muscle wasting and cardiomyopathy with conduction defects (9, 35). Moreover, deficiency or point mutations in the *Lmna* gene, encoding lamin A/C proteins, cause EDMD-like phenotypes (36-39). Conversely, KI/KI mice do not display pathological defects in skeletal muscle. This could be explained by functional redundancy between LEMD2 and other NEPs or LEM domain–containing proteins. We speculate that other NEPs may fulfill the function of LEMD2 in muscle and other tissues, as it occurs between emerin and LAP1 (40) or between emerin and the LEMD2 ortholog in *Caenorhabditis* *elegans* (41). In this regard, loss of the muscle-specific NEP NET39 in mice triggers a severe phenotype only in skeletal muscles. Moreover, a direct physical interaction between LEMD2 and NET39 has been reported in C2C12 myotubes (42). In the future, further studies will be necessary to decipher whether this interaction is functionally important. If so, this finding may explain why KI/KI mice only develop a phenotype in the heart, where NET39 is not expressed.

Regarding the pathogenic basis of *Lemd2*-associated cardiomyopathy, LEMD2 is located in the INM and has been shown to interact with both lamin A and BAF, two important chromatin regulators (13, 16). Consistent with such interactions, electron microscopy revealed a dramatic loss of transcriptionally inactive heterochromatin that is associated with the NE. The control of chromatin organization by NEPs affects gene expression (43). Accordingly, transcriptomic analysis in both *Lemd2* mouse models revealed numerous alterations in the expression of genes involved in various molecular pathways. Among them, we found strong activation of the molecular pathway orchestrated by the master regulator p53, which controls a variety of cellular processes, including the DDR and apoptosis (44, 45). Indeed, immunofluorescence analysis on cardiac sections and isolated CMs showed that the double-strand break marker γ-H2AX was present in CM nuclei of the 2 mutant models. DNA damage could be a direct consequence of NE deformations and abnormal mechanotransduction activity in *Lemd2*-deficient CMs. We hypothesize that these alterations represent a pathogenic mechanism in both *Lemd2* models. Damaged CMs also develop hypertrophy and reduced proliferation and undergo cell death. Finally, cardiac fibrosis is triggered as a consequence of apoptosis. These cellular defects culminate in pathophysiological alterations in *Lemd2* mutant mice, including DCM and AV block, which ultimately lead to sudden death. Similar pathogenic mechanisms have been described in other mouse models with mutations in NEPs and lamins (46). These findings reinforce the role of NEPs in controlling chromatin organization and suggest that common mechanisms underlie cardiomyopathies associated with different NEPs.

There are currently no available treatments for individuals with cardiomyopathy due to LEMD2 mutations. In this work, we describe an AAV-based gene therapy designed to specifically increase LEMD2 levels in CMs. Using this approach, we were able to improve cardiac morphology and function of KI/KI mice, highlighting the potential of this therapeutic strategy.

It remains to be understood how a change in one amino acid in the LEM domain of LEMD2 impairs protein stability. However, this work is the first, to our knowledge, to comprehensively study the effects of LEMD2 loss of function in the heart and the pathogenic mechanisms of LEMD2-associated cardiomyopathy. Taken together, our findings indicate that LEMD2 is essential for proper cardiac homeostasis and function through its control of nuclear stability, chromatin organization, and gene expression, and its loss of function leads to severe cardiomyopathy driven by DNA damage and p53 activation.

## Methods

### Mouse models.

For the generation of the *Lemd2* KI/KI mouse model, we designed 3 sgRNAs in the proximity of the *Lemd2* mutation and selected the most efficient one after in vitro validation: *Lemd2*-sgRNA-2, 5′-GCAGCTCTCGCCGCAACTCC-3′.

Additionally, we designed a donor template consisting of an ssODN (IDT Ultramer DNA oligos), including the pathogenic *Lemd2* mutation (c.T38>G) and a silent mutation (c.G24>A) to prevent recutting after editing, surrounded by 2 homology arms (91 nt in the 5′ arm and 36 nt in the 3′ arm): *Lemd2*-ssODN-2, 5′-GGCTGCCGGCGGGAGCAGTTCCGGGTGCGGTGCGCGCCGGGGGCGGGCGAGGGGGCGGTGTCCTGGCCATGGCCGGCCTGTCGGACCTGGAATTGCGGCGAGAGCGGCAGGCCCTGGGCTTCCAGCCAGGCCCCATCACCGA-3′.

Cas9 mRNA, *Lemd2* sgRNA, and ssODN were injected into the pronucleus of mouse zygotes. For zygote production, B6C3F1 (6 week old) female mice were treated for superovulation and mated to B6C3F1 stud males. Zygotes were isolated and transferred to M16 (Brinster’s medium for ovum culture with 100 units/mL penicillin and 50 mg/mL streptomycin). Subsequently, zygotes were injected in M2 medium (M16 medium and 20 mM HEPES) and cultured in M16 medium for 1 hour at 37°C. Injected zygotes were transferred into the oviducts of pseudo-pregnant ICR female mice.

Tail genomic DNA was extracted from F0 mice, and the correct insertion of the mutations was confirmed by Sanger sequencing. F0 mosaics were mated to C57BL/6N mice to generate mice heterozygous for the c.T38>G mutation. By intercrossing the heterozygous mice, we generated KI/KI animals. For genotyping, we used Custom TaqMan SNP Genotyping Assay (Thermo Fisher, 4332077).

For the cKO mouse model, we designed 3 sgRNAs 5′ and 3 sgRNAs 3′ of exon 1 and selected the most efficient one on each side after in vitro validation: *Lemd2*-sgRNA-51, 5′-CTCGACGCCCATCCGGAGAC-3′ and *Lemd2*-sgRNA-33, 5′-CCTTCGGGGAATGCCTGCCG-3′.

Additionally, we designed 2 ssODN (IDT Ultramer DNA Oligos) donor templates consisting of LoxP sites, surrounded by 2 homology arms (91 nt in the 5′ arm and 36 nt in the 3′ arm): *Lemd2*-ssODN-51, 5′-CTAACGCAGCGTTAGCACGTGGTAAACGTTCAATGGAATGTTGATTTACTTAATGGATGAGCTCAATGGTGTAAGAAAACCACCGCCATTAGCTAGCATAACTTCGTATAATGTATGCTATACGAAGTTATATAGAGATCCGGCCCACTTGCCAGTCTCCGGATGGG-3′ and *Lemd2*-ssODN-33, 5′-CCCACACCTGAGCCACAGGCAGGGTCAGACTCCTTTTGACAAATGAAGGCCCGGTGGTGCCAGTTTTGCTGCTACAAAGCTGAGACCACGGACTAGTATAACTTCGTATAATGTATGCTATACGAAGTTATCAGGCATTCCCCGAAGGCAGGGAAGACAAGAGGGGC-3′.

Zygotes were injected as described above.

Tail genomic DNA was extracted from F0 mice, and the correct insertion of the LoxP sites was confirmed by PCR using the following primers: *Lemd2*-5′-forward, 5′-TGTTGTTACGCCCAGAGTCTT-3′; *Lemd2*-5′-reverse, 5′-CTATGTCCGCCATGATGAAA-3′; *Lemd2*-3′-forward, 5′-AAAGCCACACGCACACTCTT-3′; and *Lemd2*-3′-reverse, 5′-GTGCCAGTTTTGCTGCTACA-3′.

F0 mosaic mice were mated to C57BL/6N mice to generate mice heterozygous for the LoxP sites. By intercrossing the heterozygous mice, we generated *Lemd2*-floxed animals. By breeding these animals with transgenic mice expressing Myh6-Cre (Jackson Laboratory, 011038), we generated *Lemd2*-cKO mice. For genotyping, we used the abovementioned primers. To validate the *Lemd2* exon 1 excision (take out), *Lemd2*-5′-forward and *Lemd2*-3′-reverse primers were used. See complete unedited blots in the supplemental material.

### Histology, immunofluorescence, and electron microscopy.

All histology was performed by staff at the Research Histo Pathology Core at University of Texas Southwestern. Hearts for routine histology were harvested from euthanized mice while still beating and allowed to pump their chambers free of blood in PBS. Subsequently, hearts were fixed overnight in 4% paraformaldehyde (PFA), dehydrated, cleared, and paraffin embedded by standard histologic procedures (47, 48). The resulting dorsoventral, 4-chamber embeds were serially sectioned on Leica RM2345 rotary microtomes at 5 μm thickness, for routine H&E and Masson’s trichrome staining. Skeletal muscle tissues were flash-frozen in a cryoprotective 3:1 mixture of Tissue Freezing Media (TFM) (Thermo Fisher Scientific, 15-183-13) and gum tragacanth (MilliporeSigma, G1128) and sectioned on a cryostat. Finally, routine H&E was performed. Images were taken using KEYENCE BZ-X700 series microscope.

For tissue immunofluorescence, heart tissues were fixed overnight at 4°C with 4% PFA prepared in PBS and cryoprotected with a sucrose gradient: 10% and 20% sucrose for 12 hours each at 4°C. Finally, tissues were embedded in TFM (Thermo Fisher Scientific, 15-183-13) and sectioned at 10 μm using a Leica CM1950 cryostat. Cryosections were air-dried for 30 minutes at room temperature, fixed in 4% PFA for 15 minutes, and washed 3 times with PBS. Antigen retrieval was then performed on an IHC-Tek steamer (IHC World, IW-1102) for 60 minutes using IHC-Tek Epitope Retrieval Solution (IHC World, IW-1100). Subsequently, samples were blocked and permeabilized using 10% goat serum/0.3% Triton X-100 in PBS with Mouse on Mouse (M.O.M.) blocking solution (Vector Laboratories, BMK-2202) for 60 minutes at room temperature. Sections were then incubated overnight at 4°C with the following primary antibodies prepared in 5% goat serum/0.3% Tween-20 in PBS: γ-H2AX (CST, 9718S, clone 20E3; 1:100), cTnT (Proteintech, 15513-1-AP; 1:100), cTnT (Thermo Fisher Scientific, MA5-12960, clone 13-11; 1:100), Ki67 (Thermo Fisher Scientific, PA5-19462, 1:200), and HCN4 (Abcam, ab32675, clone SHG 1E5; 1:50). Sections were subsequently washed with 0.01% Triton X-100 in PBS 3 times and incubated with the corresponding secondary antibodies prepared in 5% goat serum in PBS at room temperature for 1 hour: goat anti-rat Alexa Fluor 488 (Thermo Fisher Scientific, A-11006, 1:400) and goat anti-rabbit Alexa Fluor 488 (Thermo Fisher Scientific, A-11008, 1:400). After 60 minutes of secondary antibody incubation along with DAPI nuclear staining (2 mg/mL), sections were washed with PBS and mounted in Immu-Mount (Fisher, 9990412) or ProLong Gold Antifade Reagent with DAPI (Thermo Scientific, P36971) medium. Images were acquired using a Zeiss LSM 800 confocal microscope. TUNEL assay was performed using the Click-iT Plus TUNEL Assay for In Situ Apoptosis Detection Kit (Thermo Fisher Scientific, C10619) following the manufacturer’s protocol.

For electron microscopy of the whole heart, mice were perfused with 4% PFA and 1% glutaraldehyde in 0.1M sodium cacodylate buffer (pH 7.4) and stained with 1% osmium tetroxide. Samples were processed by the University of Texas Southwestern Medical Center Electron Microscopy Core facility. Images were acquired using a JEOL 1400 Plus transmission electron microscope.

### Plasmids and cloning.

The human open reading frame (ORF) of LEMD2 was purchased in pMGF196 from Addgene (97005). Subsequently, the LEMD2 ORF was subcloned into the retroviral vector pMXs-puro (Cell Biolabs, RTV-012). To obtain the c.T38>G mutation, we used the QuikChange II XL Site-Directed Mutagenesis Kit (Agilent Technologies, 200521).

### Cell culture, overexpression, and immunofluorescence.

Mycoplasma-tested C2C12 (ATCC, CRL-1772) mouse myoblasts and Platinum-E cells (Cell Biolabs, RV-101) were cultured in 10% fetal bovine serum with 1% penicillin/streptomycin in DMEM. Platinum-E cells were used for retrovirus production. Briefly, cells were transfected with FuGENE6 (Promega, E2692) per the provider’s instructions, and 15 μg of plasmid was used for 10 cm plate transfection. At 48 and 72 hours after transfection, supernatants were collected and filtered through a 0.45 μm syringe filter. C2C12 cells were infected twice with viral supernatant supplemented with polybrene (MilliporeSigma, H9268) at a final concentration of 8 μg/ml. Forty-eight hours after the first infection, cells were replaced with fresh growth media.

For cell immunofluorescence, C2C12 cells overexpressing pMXs-puro-*LEMD2* and pMXs-puro-*LEMD2* c.T38>G were differentiated into myotubes for 5 days in DMEM with 2% horse serum. Subsequently, cells were fixed in 4% PFA for 15 minutes, washed 3 times with PBS, permeabilized with 0.3% Triton X-100 for 20 minutes, and blocked with 5% bovine serum albumin (BSA) for 30 minutes. The following primary antibodies were used in blocking solution for 2 hours at room temperature: LEMD2 (MilliporeSigma, HPA017340; 1:500). Sections were subsequently washed with PBS and stained with the corresponding secondary antibodies: goat anti-rabbit Alexa Fluor 555 (Thermo Fisher Scientific, A-27039). After secondary antibody incubation, sections were washed with PBS, incubated with DAPI at room temperature for 10 minutes, and washed twice with PBS before mounting. Images were obtained using a Zeiss LSM 800 confocal microscope.

### Western blot analysis.

Flash-frozen hearts were pulverized using a tissue crusher and protein was isolated in RIPA buffer (MilliporeSigma, R0278) containing protease and phosphatase inhibitors (Roche, 04693159001 and 04906837001). To break genomic DNA, samples were sonicated using Bioruptor Pico (Diagenode) for 10 cycles of 30-second sonication on and 30-second sonication off. Subsequently, samples were centrifuged at 12,000*g* for 20 minutes at 4°C to pellet cell debris. Protein concentration was determined by BCA assay (Thermo Fisher, 23225), and equal amounts of protein among samples were used for regular Western blot and transferred to polyvinylidene fluoride membrane (Millipore, IPVH00010).

Blocking and antibody incubation were performed in 5% milk or 5% BSA in TBS-Tween 0.1%. The following primary antibodies were used: LEMD2 (MilliporeSigma, HPA017340, 1:500) and GAPDH (MilliporeSigma, MAB374, clone 6C5; 1:500). Horseradish peroxidase–conjugated (HRP-conjugated) secondary antibodies were used (Bio-Rad, 1706515 and 1706516, 1:5000). Immunodetection was performed using Western Blotting Luminol Reagent (Santa Cruz Biotechnology Inc., sc2048) using a SRX-101A film processor (Konica Minolta) and ChemiDoc MP Imaging system (Bio-Rad). See complete unedited blots in the supplemental material.

### CM isolation.

Isolation of CMs was performed as previously described (49). Briefly, hearts were digested with 2.4 mg/ml collagenase type II in perfusion buffer (120 mM NaCl, 14.7 mM KCl, 0.6 mM KH_2_PO_4_, 0.6 mM Na_2_HPO_4_, 1.2 mM MgSO_4_, 10 mM Na-HEPES pH=7.0, 4.6 mM NaHCO_3_, 30 mM taurine, 10 mM BDM, 5.5 mM glucose) via Langendorff retroaortic perfusion. After digestion, atria and valves were removed and ventricular tissue alone was gently triturated in stop buffer (perfusion buffer with 10% fetal bovine serum with 12.5 μM CaCl_2_), then filtered through a 250-μm nylon mesh. For evaluation of CM size and morphology, CMs were fixed in 2% PFA for 15 minutes by adding an equal volume of 4% PFA, centrifuged at 300*g*, permeabilized with 0.3% Triton X-100 for 20 minutes, and blocked with 5% BSA for 30 minutes. After centrifugation and resuspension in 5% BSA, CMs were stained with anti-ACNT2 (Sigma-Aldrich, A7811, clone EA-53; 1:500) and goat anti-mouse Alexa Fluor 488 (A-21121) using standard procedures. Cells were coverslipped with ProLong Gold antifade reagent with DAPI (Thermo Scientific, P36971). The area, length and width of CMs were analyzed with ImageJ (NIH). Length was taken at the longest line parallel to the sarcomere axis and width at the longest line perpendicular to the sarcomere axis; area was calculated based on the entire cell outline, and approximately 110 CMs were analyzed per sample.

For neonatal CM isolation, we used the Mouse/Rat Cardiomyocyte Isolation Kit (Cellutron Life Technologies, NC-6031) following the manufacturer’s instructions. After isolation, cells were plated on collagen and laminin–coated glass-bottom plates and kept in culture at 37°C and 5% CO_2_ for at least 48 hours. For immunostaining, CMs were fixed in 4% PFA for 10 minutes, permeabilized with 0.3% Triton X-100 for 10 minutes, and blocked with 10% goat serum for 30 minutes. CMs were stained with γ-H2AX (CST, 9718S, clone 20E3; 1:200), cTnT (Thermo Fisher Scientific, MA5-12960, clone 13-11; 1:200), cardiac troponin I (Abcam, ab47003, 1:200), lamin B1 (Santa Cruz Biotechnology Inc., sc-374015, clone B-10; 1:50), goat anti-rabbit Alexa Fluor 488 (Thermo Fisher Scientific, A-11008, 1:400), and goat anti-mouse IgG1 Alexa Fluor 555 (Thermo Fisher Scientific, A-21127, 1:400) on 3% goat serum using standard procedures. Nuclei were stained with 16.2 mM Hoechst 33342 solution (Thermo Fisher Scientific, H3570, 1:1500). TUNEL assay was performed using the Click-iT Plus TUNEL Assay for In Situ Apoptosis Detection Kit (Thermo Fisher Scientific, C10619) following the manufacturer’s protocol. Images were taken using a KEYENCE BZ-X700 series microscope and a Zeiss LSM 800 confocal microscope.

### CM confinement experiments.

A 6-well static cell confiner device (4D cell) was used to mechanically compress isolated CMs. The device employs micropillars and PDMS pistons to compress cells in the vertical axis, generating mechanical stretch (34). After isolation, CMs were cultured for at least 48 hours and then compressed for 1 hour under a pillar length of 20 μm to induce stretching. After compression, cells were processed for downstream applications.

### CM contractility and calcium transients.

The isolation of CMs was performed as previously described (49). Briefly, following collagenase perfusion and filtering of the cell suspension through nylon mesh as described above, cells were centrifuged at 100*g* for 1 minute at room temperature, the supernatant was decanted, and cells were resuspended in 10 ml stop buffer (perfusion buffer containing 10% of FBS) and recentrifuged. Cells were successively resuspended in 10 ml stop buffer containing 100 μM, 400 μM, and 900 μM CaCl_2_ with 2-minute delay before recentrifugation. Cells were resuspended in stop buffer containing 1.2 mM CaCl_2_ with 2 μg/ml Fura 2-AM (Thermo Fisher Scientific, F1221) in the dark for 5 minutes at room temperature, then washed once in stop buffer with 1.2 mM CaCl_2_ to remove excess label. The loaded cells were then allowed to settle to the bottom of a chamber with coverslip base, which was mounted on a Motic inverted fluorescence microscope. CM contractility and calcium dynamics measurements were performed using a stepper-switch IonOptix Myocyte Calcium and Contractility System. Cells were electrically paced at 1 Hz with a 5 ms pulse of 20 volts. Sarcomere length and shortening were measured using a Fourier transform of CM *Z*-line patterns under phase contrast optics using a switching rate of 100 Hz. Fura2 calcium transients were captured simultaneously, using the ratio of Fura2 fluorescence emission at 340/380 nm at a switching rate of 1,000 Hz. Offline data measurements were performed using IonWizard 6.0 analysis software. Cells displaying asynchronous contractility, excessive blebbing/dysmorphology, and abnormally high or low shortening fraction or calcium amplitude were ignored for acquisition. No preparation of cells was left for more than 10 minutes before being replaced with a fresh batch of cells.

### Bulk RNA-Seq analysis.

Flash-frozen cardiac samples were homogenized in 1 ml of TRIzol (Thermo Fisher Scientific, 15596026) in Precellys Evolution (3 cycles × 20 seconds at 6800 rpm). RNA was isolated using the RNeasy Micro Kit (QIAGEN, 74004) per the provider’s instructions. RNA-Seq libraries (*n* = 3 mice per genotype) were prepared using the KAPA mRNA HyperPrep (Kapa Biosystems, kk8580) Kit following the manufacturer’s specifications. High-output 75-cycle single-ended sequencing was performed at the CRI Sequencing Facility at University of Texas Southwestern Medical Center using a NextSeq500 sequencer.

### Bioinformatics.

RNA-Seq analyses were conducted in R (version 3.3.2) and Python (version 3.5.4). Trim Galore (https://www.bioinformatics.babraham.ac.uk/projects/trim_galore/) was used for quality and adapter trimming. The mouse reference genome sequence and gene annotation data, mm10, were downloaded from Illumina iGenomes (https://support.illumina.com/sequencing/sequencing_software/igenome.html). The qualities of RNA-Seq libraries were estimated by mapping the reads onto mouse transcript and ribosomal RNA sequences (Ensembl release 89) using Bowtie (version 2.3.4.3) (50). STAR (version 2.7.2b) (51) was employed to align the reads onto the mouse genome, SAMtools (version 1.9) (52) was employed to sort the alignments, and the HTSeq Python package (53) was employed to count reads per gene. The DESeq2 R Bioconductor package (54) was used to normalize read counts and identify DE genes, using FDR-adjusted *P* values (Benjamini-Hochberg method) of 0.05 as cutoffs. Upstream regulator analysis was based on a custom script for identifying transcription factors that regulate DE genes. Enrichment analysis of gene sets was performed using Metascape (https://metascape.org/) with the supply of upregulated or downregulated DEGs and using *P* < 0.01 as a cutoff.

### GSEA.

GSEA was performed using GSEA software (55, 56). For the analysis, we provided the software with DEGs and selected 1,000 permutations and MSigDB.v7.4chip as platform. *P* < 0.05 was used as a cutoff.

### RT-qPCR analysis.

Total RNA was extracted from whole hearts using TRIzol (Thermo Fisher Scientific, 15596026) and the RNeasy Mini Kit (QIAGEN, 74104) and reverse transcribed using iScript Reverse Transcription Supermix (Bio-Rad) with random primers. The quantitative PCRs (qPCRs) were assembled using KAPA SYBR Fast qPCR Master Mix (KAPA, KK4605). Assays were performed using a QuantStudio 5 Real-Time PCR machine (Applied Biosystems). Expression values were normalized to 18S or Gapdh mRNA and were represented as fold-change. Oligonucleotide sequences of qPCR primers are listed in Supplemental Table 1.

### Transthoracic echocardiography.

Cardiac function was evaluated by 2D transthoracic echocardiography on conscious mice using a VisualSonics Vevo2100 Imaging System. Images were acquired as 2D and M-mode (left parasternal long and short axes), and measurements were averaged from 3 consecutive heartbeats of M-mode tracings. M-mode tracings were used to measure LVID at end diastole (LVIDd) and end systole (LVIDs). FS was calculated according to the following formula: FS (%) = [(LVIDd – LVIDs)/LVIDd] × 100. EF was calculated according to the following formula: EF (%) = (LVEDV – LVESV)/LVEDV × 100, where LVESV indicates LV end systolic volume and LVEDV indicates LV end diastolic volume. All measurements were performed by an experienced operator blinded to the study.

### Electrocardiography.

Mice were anesthetized with 1.5% isoflurane in O_2_ via face mask (following induction in a chamber containing 5% isoflurane). Rectal temperature was continuously monitored and maintained within 37 ± 0.3°C using a heat pad and heat lamp. The surface ECG (lead II) was recorded using 2 tiny alligator clip electrodes, contacting the skin of the mouse at the upper and lower front of the chest. The signal was acquired for about 1 minute using Chart (version 4.2.3) software. The ECG was recorded and analyzed using a digital acquisition and analysis system (Power Lab/4SP; ADInstruments). QTc duration was calculated using the Bazett formula (QTc = QT/√RR). All measurements were performed by an experienced operator blinded to the study.

For the β-adrenergic stimulation experiment, 3 administrations of ISO dissolved in 0.9% NaCl were delivered, starting with a dose of 40 mg/kg of body weight followed by 2 consecutive injections at 80 mg/kg of body weight 5 and 15 minutes after the first dose (57, 58). ECG was recorded for 35 minutes after the first injection.

### Lemd2 gene therapy.

The mouse LEMD2 ORF was purchased from Addgene (plasmid 120245) and cloned into the previously generated AAV9 vector under the control of the cTnT promoter (59). AAVs were prepared by the Boston Children’s Hospital Viral Core (Boston, Massachusetts, USA), as previously described (60). The i.p. injection of P4 KI/KI mice was performed using an ultrafine needle (31 gauge) with 80 μl of saline solution containing the AAV9-*Lemd2* viruses (5 × 10^13^ vg per kg). The AAV9-*Lemd2* treatment was unblinded for mouse genotypes, and data were compared with untreated WT and KI/KI groups shown in Figure 2, A–F, that were not assessed contemporaneously.

### Data availability.

All data presented in this study are available in the main text or the supplemental material. RNA-Seq data generated during this study were deposited in the NCBI’s Gene Expression Omnibus database (GEO GSE194218).

### Statistics.

Data are presented as mean ± SEM. Prism software was used for statistical analysis and data plotting. No data were excluded. For physiological, histological, and cellular experiments, statistical analysis was performed using 2-tailed, unpaired *t* tests or 1-way ANOVA with Holm-Šidák correction for multiple comparisons, as indicated in each figure legend. *P* < 0.05 was considered significant, and statistically significant differences are shown with asterisks. Normal distribution was assumed for all variables. For genome-wide analysis, a fold change greater than 2 and adjusted *P* < 0.05 were used. Sample sizes are indicated in each figure legend. Fiji (ImageJ) was utilized for image analysis, calculation of nuclear solidity (area/convex area), and fibrosis quantification (61).

### Study approval.

All animal procedures were approved by the Institutional Animal Care and Use Committee at the University of Texas Southwestern Medical Center.

## Author contributions

XMC, RBD, NL, and ENO designed the experiments and overall study and wrote the manuscript. XMC, ARM, and JRM generated the mouse models. XMC and PG performed the experiments. FW and LX did the bioinformatics analysis. All authors discussed the results and participated in the article preparation and editing.

## Supplementary Material

Supplemental data

## Version 1 11/15/2022

Electronic publication
