# Supplementary material for: Loss of function of the nuclear envelope protein LEMD2 causes DNA damage–dependent cardiomyopathy
Source: J Clin Invest. 2022 Nov 15;132(22):e158897. doi: 10.1172/JCI158897 (PMC9663152; doi:10.1172/JCI158897)

Loss of function of the nuclear envelope protein LEMD2 causes DNA damage-dependent cardiomyopathy

Xurde M. Caravia[1,2], Andres Ramirez-Martinez[1,2], Peiheng Gan[1,2], Feng Wang[3], John R. McAnally[1,2], Lin Xu[3], Rhonda Bassel-Duby[1,2], Ning Liu[1,2] & Eric N. Olson[1,2,*]

[1]Department of Molecular Biology, Hamon Center for Regenerative Science and Medicine, University of Texas Southwestern Medical Center, Dallas, TX, USA. [2]Senator Paul D. Wellstone Muscular Dystrophy Specialized Research Center, University of Texas Southwestern Medical Center, Dallas, TX, USA. [3]Quantitative Biomedical Research Center, Department of Population & Data Sciences and Department of Pediatrics, University of Texas Southwestern Medical Center, Dallas, TX, USA.

*Corresponding author: Eric N. Olson, Ph.D.; Mailing address: UT Southwestern Medical Center. Department of Molecular Biology. 6000 Harry Hines Blvd. Dallas, TX 75390-9148; Phone: +1 214-648-1187; Email: eric.olson@utsouthwestern.edu

Supplemental information

Supplemental figures

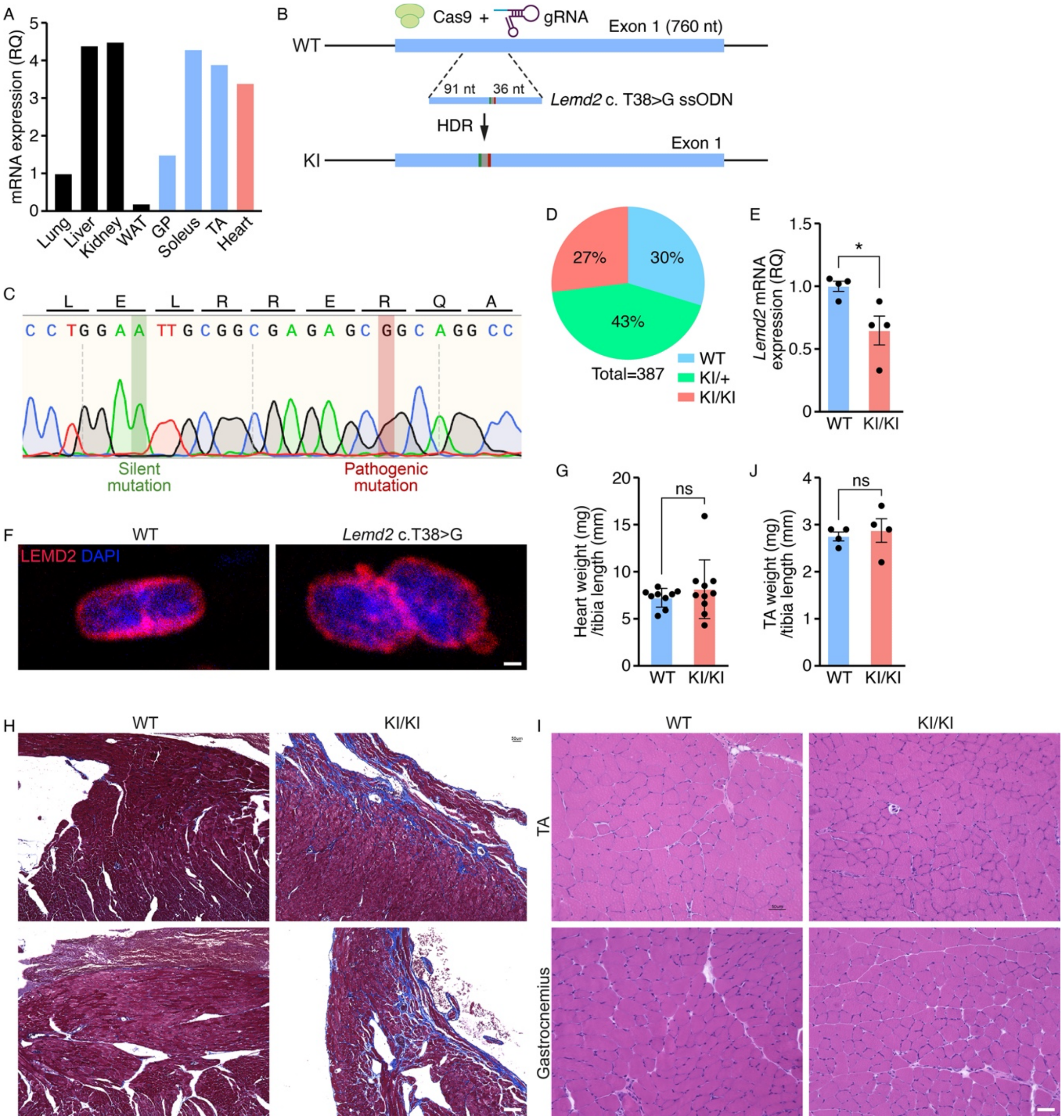

Supplemental Figure 1: Generation and characterization of the KI/KI mouse model. A) Lemd2 mRNA expression in mouse tissues normalized to lung (GP, gastrocnemius-plantaris; TA, tibialis anterior; WAT, white adipose tissue). **B)** Schematic of the CRISPR/Cas9 strategy to generate the KI/KI mice. **C)** Sanger sequencing of a KI/KI mouse. **D)** Genotype frequency distribution seven days after birth (P7) of WT, KI/+

and KI/KI mice from heterozygous breeding (387 mice, p= non-significant; Chi-square test). **E)** *Lemd2* mRNA expression relative to *Gapdh* in hearts from 2-month-old WT (n= 4) and KI/KI (n= 4) mice (*p<0.05; two-tailed unpaired t test). **F)** Immunofluorescence showing the localization of LEMD2 WT and LEMD2 c.T38>G after their retroviral overexpression in C2C12 myotubes (scale bar: 10 μm). **G)** Heart weight / tibia length ratio in WT and KI/KI mice (ns (non-significant) p>0.05; two-tailed unpaired t test). **H)** Masson Trichrome staining of hearts from WT and KI/KI mice (scale bar: 50 μm). **I)** H&E staining of tibialis anterior (TA) and gastrocnemius muscle groups from WT and KI/KI mice (scale bar: 50 μm). **J)** Tibialis anterior (TA) weight / tibia length ratio in WT and KI/KI mice (ns (non-significant) p>0.05; two-tailed unpaired t test).

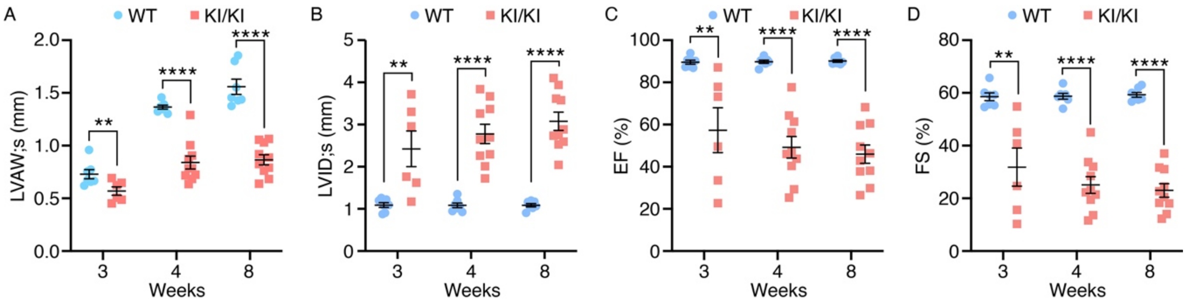

Supplemental Figure 2: KI/KI mice develop DCM. Echocardiographic analysis of structural and functional parameters in systolic hearts from WT and KI/KI mice: **A)** Systolic left ventricular anterior wall (LVAW;s) thickness (3w **p<0.01, 4w ****p<0.0001, 8w ****p<0.0001, two-tailed unpaired student t test). **B)** Systolic left ventricular internal diameter (LVID;s) (3w **p= 0.01, 4w ****p<0.0001, 8w ****p<0.0001, two-tailed unpaired student t test), **C)** Ejection fraction (EF) (3w **p<0.01, 4w ****p<0.0001, 8w ****p<0.0001, two-tailed unpaired student t test) and **D)** Fractional shortening (FS) (3w **p<0.01, 4w ****p<0.0001, 8w ****p<0.0001, 7 WT and 6 KI/KI mice for the 3w comparison and 7 WT and 10 KI/KI mice for the 4w and 8w comparisons; two-tailed unpaired student t test).

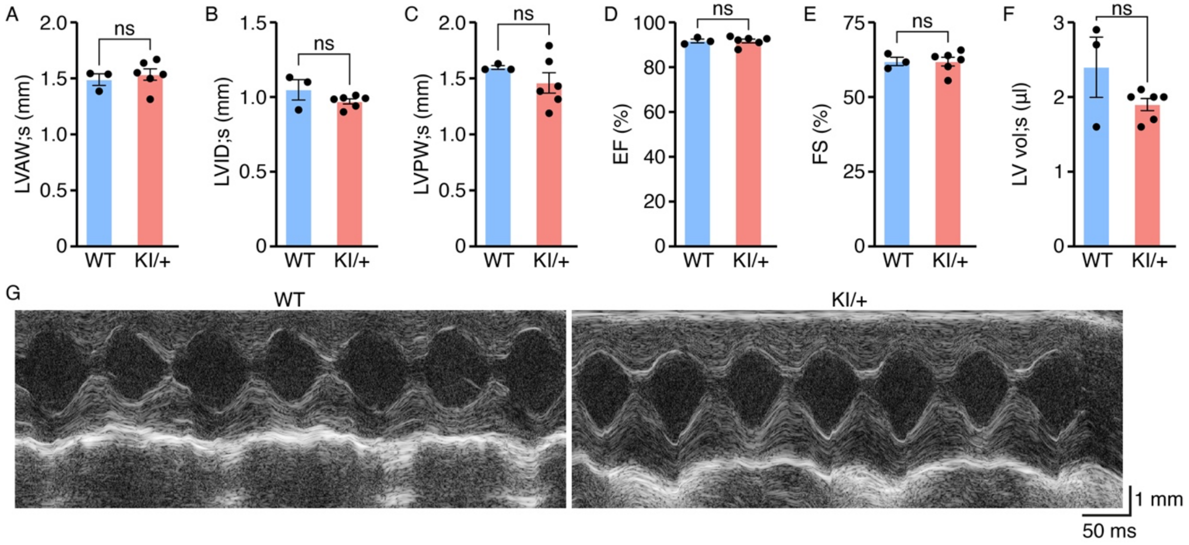

Supplemental Figure 3: Mice heterozygous for the *Lemd2* c.T38>G (KI/+) mutation have a preserved cardiac function. Echocardiographic analysis of structural and functional parameters in systolic hearts from 2-month-old WT (n=3) and KI/+ mice: **A)** Systolic eft ventricular anterior wall (LVAW;s) thickness, **B)** Systolic left ventricular internal diameter (LFID;s), **C)** Systolic left ventricular posterior wall (LVPW;s) thickness, **D)** Ejection fraction (EF), **E)** Fractional shortening (FS) and **F)** Left ventricle volume (n=6). **G)** Representative transthoracic M-mode echocardiographic tracings of 2-month-old WT and KI/+ mice. (ns (non-significant) $p>0.05$; two-tailed unpaired t test for all the comparisons).

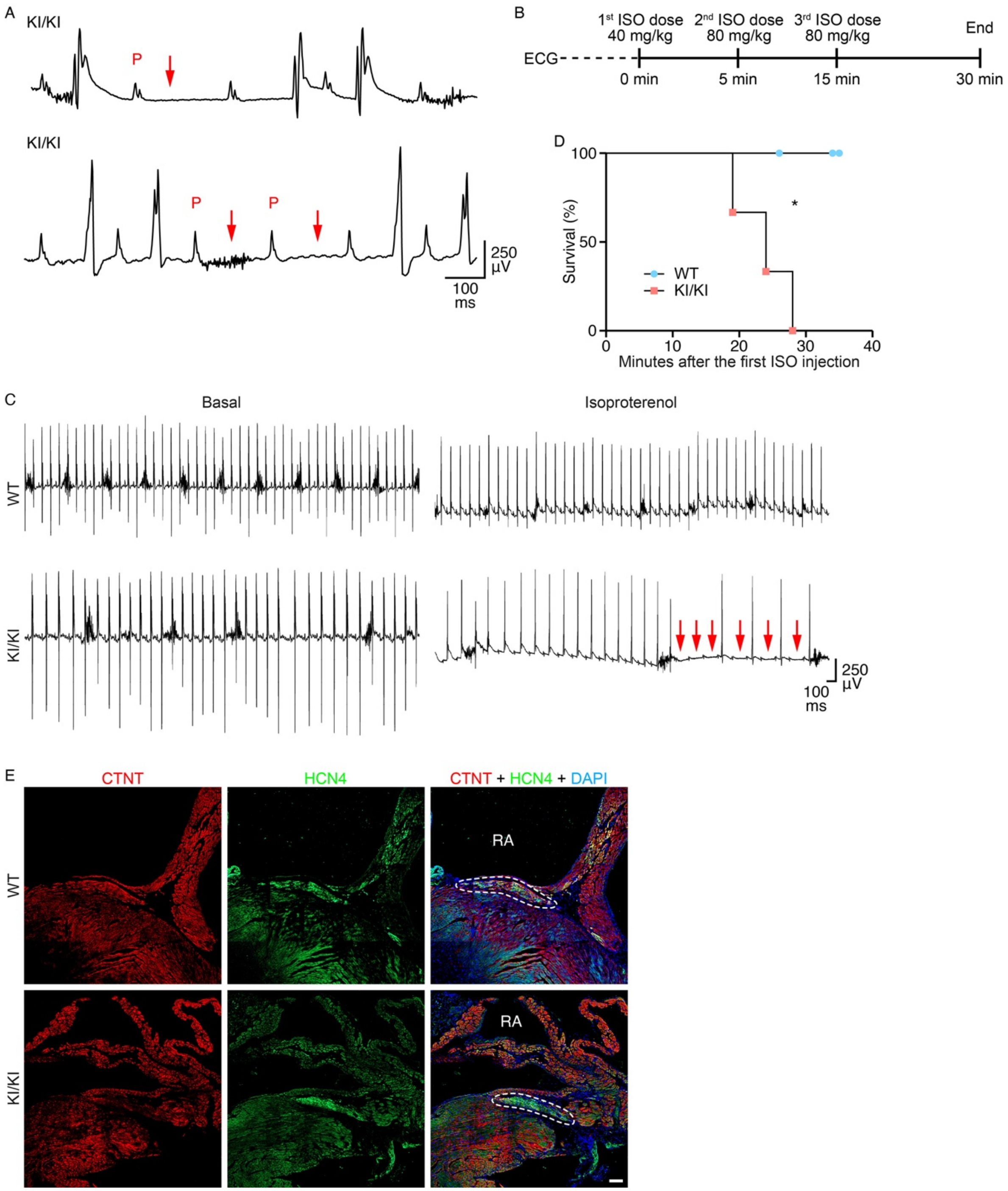

Supplemental Figure 4: KI/KI mice show cardiac electrical abnormalities. A) ECG of two 2-month-old KI/KI mice showing the type II AV block (arrows indicate the absence of the QRS complex). **B)** Schematic of the isoproterenol (ISO) administration protocol. **C)** Representative ECG from mice at 4-5 months old before (basal) and after ISO administration (arrows indicate the absence of the QRS complex). **D)** Survival curve of

WT (n=3) and KI/KI (n=3) mice after the first ISO administration (Log-rank (Mantel-Cox) test; *p<0.05). **E)** Immunostaining of cardiac sections from WT and KI/KI mice against the CM marker cardiac troponin T (cTnT) and the cardiac conduction system-specific marker HCN4. (White lines mark the AV node; RA: right atrium; scale bar: 100 μm).

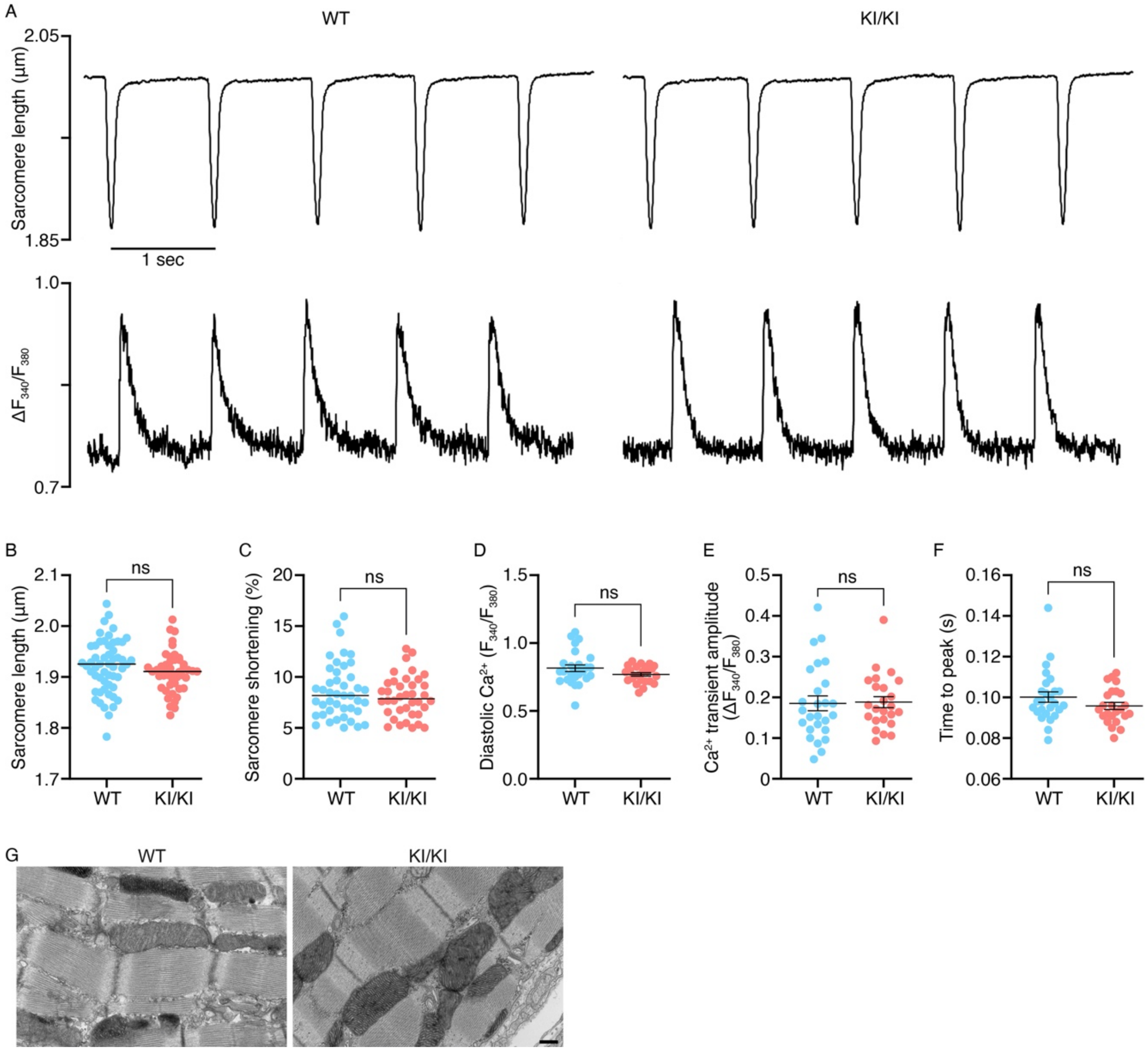

Supplemental Figure 5: Structure, sarcomere contractility and calcium dynamics in KI/KI isolated CMs. A) Representative sarcomere contraction (top) and calcium transients (bottom) of WT and KI/KI CMs. **B)** Sarcomere length in CMs from WT (n=4) and KI/KI (n=3) mice. **C)** Percentage of sarcomere fractional shortening in CMs from WT (n=4) and KI/KI (n=3) mice. **D)** Ratio of diastolic calcium levels (F_{340}/F_{380}) measured using the Fura-2 dye in CMs from WT (n=4) and KI/KI (n=3) mice. **E)** Calcium transient amplitude (from basal level to peak) measured using the Fura-2 dye in CMs from WT (n=4) and KI/KI (n=3) mice. **F)** Time to calcium peak measured using the Fura-2 dye in CMs from WT (n=4) and KI/KI (n=3) mice**. G)** Representative transmission EM pictures of the sarcomere structure of 3-month-old WT and KI/KI hearts (scale bar: 2 μm). (ns (non-significant) $p>0.05$; two-tailed unpaired t test for all the comparisons).

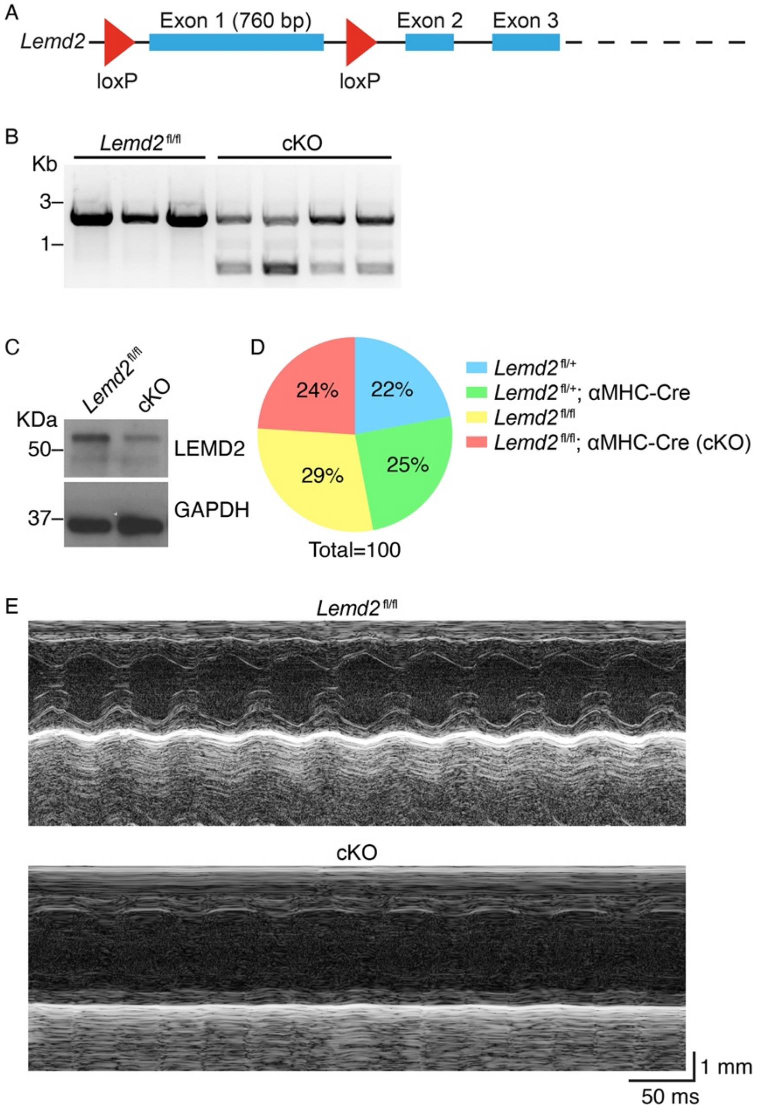

Supplemental Figure 6: Generation and characterization of cKO mice. A) Scheme showing the *Lemd2*-floxed allele**. B**) PCR showing the excision of the *Lemd2*-floxed allele (first exon) in cardiac tissue after the expression of the recombinase Cre under control of the αMHC promoter (floxed band=1.5 Kb; KO band=80bp). **C)** Western blot analysis showing the expression of both LEMD2 cardiac isoforms in heart protein lysates from *Lemd2* $^{fl/fl}$ and cKO mice. **D)** Genotype frequency distribution one day after birth (P1) of mice from *Lemd2* $^{fl/+}$ αMHC-Cre x *Lemd2* $^{fl/fl}$ breeding (100 mice, p= non-significant; Chi-square test). **E)** Transthoracic M-mode echocardiographic tracings of P7 *Lemd2* $^{fl/fl}$ and cKO mice.

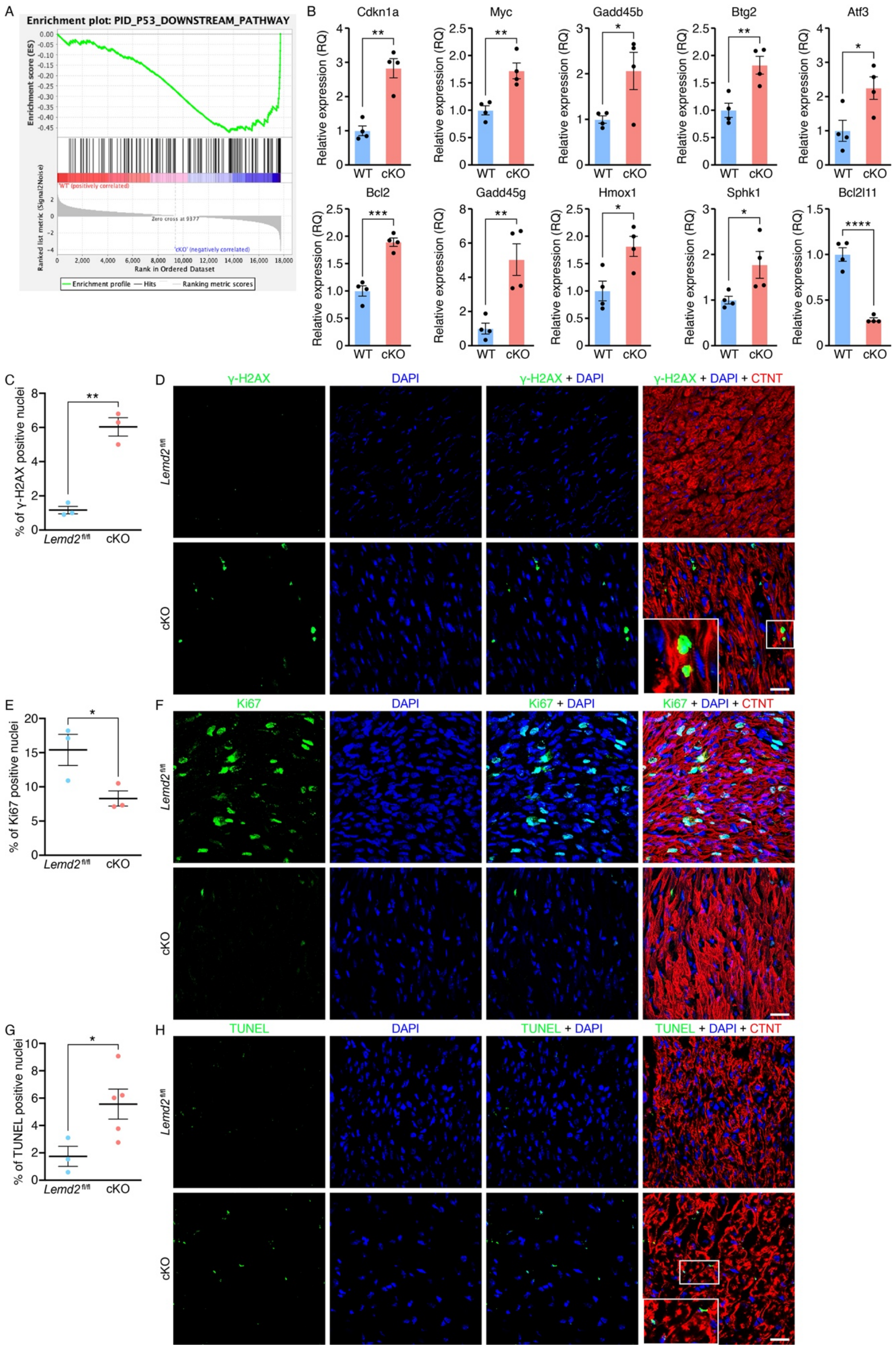

A
Enrichment plot: PID_P53_DOWNSTREAM_PATHWAY
Enrichment score (ES)
'WT' (positively correlated)
Zero cross at 9377
'cKO' (negatively correlated)
Ranked list metric (Signal2Noise)
Rank in Ordered Dataset
Enrichment profile
Hits
Ranking metric scores
B
Cdkn1a
Myc
Gadd45b
Btg2
Atf3
Bcl2
Gadd45g
Hmox1
Sphk1
Bcl2l11
Relative expression (RQ)
WT
cKO
C
% of γ-H2AX positive nuclei
Lemd2 fl/fl
cKO
D
γ-H2AX
DAPI
γ-H2AX + DAPI
γ-H2AX + DAPI + CTNT
E
% of Ki67 positive nuclei
F
Ki67
Ki67 + DAPI
Ki67 + DAPI + CTNT
G
% of TUNEL positive nuclei
H
TUNEL
TUNEL + DAPI
TUNEL + DAPI + CTNT

Supplemental Figure 7. Activation of p53 signaling pathway, DNA damage and cellular apoptosis in cKO mice. A) GSEA plot showing the enrichment of genes related p53 downstream pathway in cKO mice. Note that the enrichment score (green line) deviates from 0 in the right part of the plot, indicating that those genes are enriched in the cKO mice (3 mice per genotype). **B)** mRNA expression, normalized to 18S, of genes related to p53 signaling and DDR in WT and cKO hearts (4 mice per genotype; Cdkn1a **$p<0.01$, Myc **$p<0.01$, Gadd45b *$p<0.05$, Btg2 **$p<0.01$, Atf3 *$p<0.05$, Bcl2 ***$p<0.001$, Gadd45g **$p<0.01$, Hmox1 *$p<0.05$, Sphk1 *$p<0.05$ and Bcl2l11 ****$p<0.0001$; two-tailed unpaired t test). **C)** Quantification of the percentage of nuclei positive for γ-H2AX staining in *Lemd2* $^{fl/fl}$ and cKO hearts (3-4 mice per genotype, more than 100 nuclei per mouse, **$p<0.01$ two-tailed unpaired t test). **D)** Representative pictures of γ-H2AX staining in cardiac sections from P5 *Lemd2* $^{fl/fl}$ and cKO mice (scale bar: 20 μm). **E)** Quantification of the percentage of nuclei positive for Ki67 staining in *Lemd2* $^{fl/fl}$ and cKO hearts (3 mice per genotype, more than 500 nuclei per mouse, *$p=0.048$ two-tailed unpaired t test). **F)** Representative pictures of Ki67 staining in cardiac sections from P5 *Lemd2* $^{fl/fl}$ and cKO mice (scale bar: 20 μm). **G)** Quantification of the nuclei positive for TUNEL staining in *Lemd2* $^{fl/fl}$ and cKO mice (3-4 mice per genotype, more than 100 nuclei per mouse, **$p=0.0493$ two-tailed unpaired t test). **H)** Representative pictures of TUNEL staining in cardiac sections from P5 WT and cKO mice (scale bar: 20 μm).

Supplemental Table 1. Sequences of the primers used for RT-qPCR gene expression analysis.

Primer ID	Sequence (5' -> 3')
Atf3_Fw	AAGACTGGAGCAAAATGATG
Atf3_Rv	GTCAGGTTAGCAAAATCCTC
Bcl2_Fw	ATGACTGAGTACCTGAACC
Bcl2_Rv	ATATAGTTCCACAAAGGCATC
Bcl2l11_Fw	ACGAGTTCAACGAAACTTAC
Bcl2l11_Rv	TAGATCCTGTCAATGCCTTC
Btg2_Fw	CTGACCGATCATTACAAACAC
Btg2_Rv	AGACACTTCATAGGGATCAAC
Gadd45g_Fw	CTGCAGATCCATTTCACG
Gadd45g_Rv	TTAGGATTCGAAATGAGGATG
Hmox1_Fw	CATGAAGAACTTTCAGAAGGG
Hmox1_Rv	TAGATATGGTACAAGGAAGCC
Sdc1_Fw	CTTCTGTCATCAAAGAGGTTG
Sdc1_Rv	CAAAGGTGAAGTCTTGTTCTC
Sphk1_Fw	GGTACTCTCATCTCGACTTC
Sphk1_Rv	GCCAGATTTTTAGCTTCCAG
Cdkn1a_Fw	ACCTGATGATACCCAACTAC
Cdkn1a_Rv	CTGTGGCACCTTTTATTCTG
Myc_Fw	TTTTGTCTATTTGGGGACAG
Myc_Rv	CATAGTTCCTGTTGGTGAAG
Gadd45b_Fw	TGCAATCTTCTTTTTACCCC
Gadd45b_Rv	CAAGAGCAAAGTACAAGTCC
Lemd2_Fw	GACTGTGAGAGAAMACAGATG
Lemd2_Rv	CACATTAGCTATGTACTCCTG
Gapdh_Fw	AGGTCGGTGTGAACGGATTTG
Gapdh_Rv	TGTAGACCATGTAGTTGAGGTCA
18S_Fw	CCATCCAATCGGTAGTAGCG
18S_Rv	GTAACCCGTTGAACCCCATT

Unedited versions of all gel and blot images included in the manuscript entitled "Loss of function of the nuclear envelope protein LEMD2 causes DNA damage-dependent cardiomyopathy"

Lanes highlighted in red are included in the figures.

- Full unedited protein blot for Figure 1C:

LEMD2

WT KI/KI

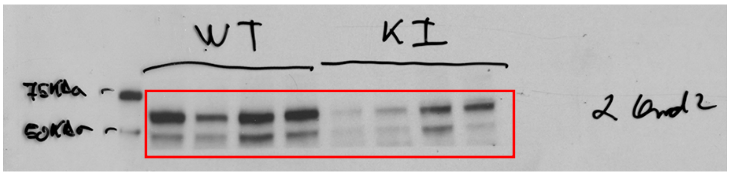

GAPDH

WT KI/KI

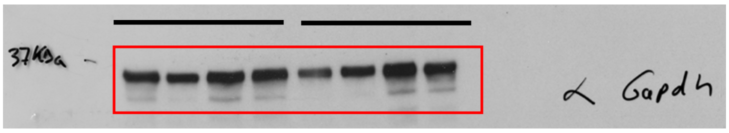

- Full unedited protein blot for Figure 7L:

LEMD2

WT KI/KI KI/KI; AAV9-*Lemd2*

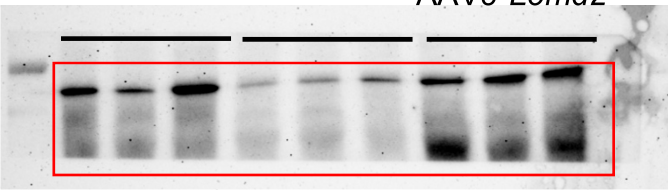

GAPDH

WT KI/KI KI/KI; AAV9-*Lemd2*

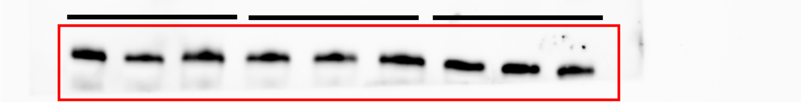

- Full unedited DNA gel for Supplemental Figure 6B:

Lemd2 (DNA excision)

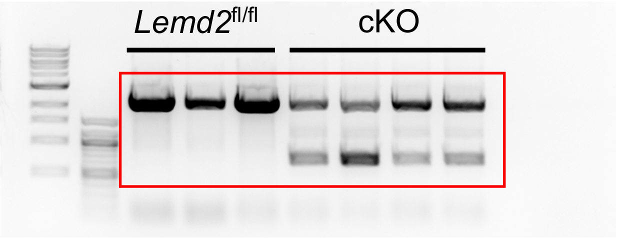

- Full unedited protein blot for Supplemental Figure 6C:

LEMD2

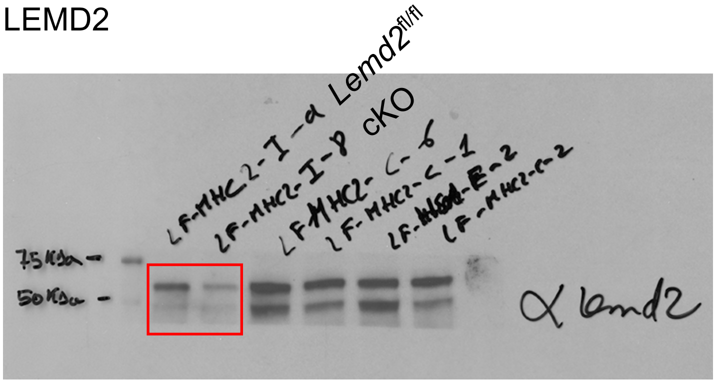

GAPDH

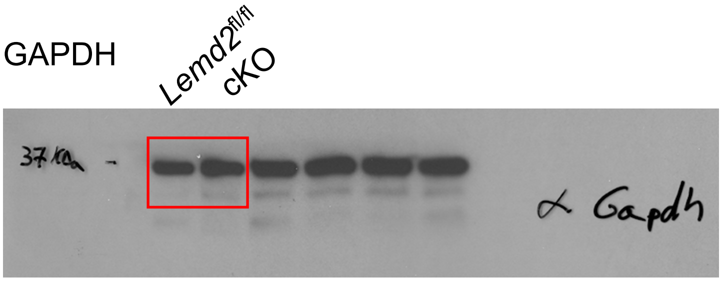

Supplement: Supplemental data [file jci-132-158897-s098.pdf]
